# Numerical and Experimental Study of the Effects of Water-to-Cement Ratio and Temperature on Cement-Based Materials Under Sulfate Attack

**DOI:** 10.3390/ma19194218

**Published:** 2026-10-03

**Authors:** Tongning Cao, Ze Chen, Chenyun Yu, Bin Peng, Yuncheng Wang, Fengjuan Wang, Jinyang Jiang

**Affiliations:** 1 State Key Laboratory of Engineering Materials for Major Infrastructure, Southeast University, Nanjing 211189, China; 2 School of Materials Science and Engineering, Southeast University, Nanjing 211189, China; 3 Engineering Management Center, China State Railway Group Co., Ltd., Beijing 100844, China; 4 China Railway Engineering Consulting Group Co., Ltd., Beijing 100071, China

**Keywords:** water-to-cement ratio, temperature, sulfate attack, transport–reaction model, quantitative characterization

## Abstract

The water-to-cement ratio and temperature are key factors governing ionic transport in cement-based materials. However, quantitative descriptions of their coupled effects on sulfate ion transport–reaction processes remain limited. In this study, a coupled transport–reaction model is developed to simulate sulfate ingress in cementitious materials subjected to external sulfate attack. The predictive capability of the model is validated through a series of well-designed experiments, and the distributions of sulfate ion concentration within the materials under different water-to-cement ratios and temperature conditions are systematically analyzed. The results demonstrate good agreement between model predictions and experimental measurements, indicating that increases in both water-to-cement ratio and temperature significantly accelerate sulfate diffusion and intensify chemical reactions between sulfate ions and cement hydration products, thereby promoting material degradation. These findings highlight that the coupled effects of water-to-cement ratio and temperature should be explicitly considered in durability assessment and numerical modeling of external sulfate attack in cement-based materials to improve predictive accuracy.

## 1. Introduction

Sulfate attack remains a major durability concern for cement-based materials exposed to sulfate-rich environments. Its development involves sulfate transport through the pore network, chemical reactions with cement hydrates, and the accumulation of secondary reaction products. The water-to-cement ratio is a primary parameter governing pore structure development in cementitious materials [1,2,3,4,5]. A lower water-to-cement ratio generally produces a denser and less connected pore network, thereby restricting sulfate transport and delaying the accumulation of expansive reaction products. In contrast, higher water-to-cement ratios promote pore connectivity, enhance ionic diffusivity, and accelerate sulfate penetration and damage evolution [6,7]. Once sulfate reaches the aluminate phases, it can react with these phases to form secondary ettringite (AFt). When AFt grows within confined pores under sufficient supersaturation, the resulting crystallization pressure can induce expansion and microcracking [8]. Experimental observations on cement paste have also associated AFt precipitation with pore filling, pore blockage, microcracking, and changes in transport behavior [9]. These feedbacks among sulfate transport, phase evolution, and material damage have become important components of reactive transport descriptions of sulfate attack [10]. Recent reviews of sulfate resistance testing further show that the observed deterioration depends on specimen scale, permeability, binder composition, and exposure conditions [11]. Susceptibility to sulfate attack therefore depends not only on pore connectivity but also on the availability of reactive hydrates and the potential amount of AFt that can form [12].

Temperature also plays a critical role in sulfate attack by affecting both transport properties and reaction kinetics. Elevated temperatures increase ionic mobility and accelerate sulfate-induced reactions with hydration products, thereby modifying both the rate and mechanisms of degradation [13,14,15,16,17]. These observations indicate that sulfate attack is governed by a strongly coupled transport–reaction process, in which water-to-cement ratio and temperature interact in a non-independent manner. Experiments under combined temperature and sulfate exposure have shown that AFt formation, pore structure, and mechanical performance may evolve differently during the early and later stages of exposure [18]. However, despite their recognized importance, quantitative and mechanistic descriptions of the coupled effects of water-to-cement ratio and temperature on sulfate transport and material degradation remain limited [19,20].

Experimental investigations have traditionally focused on macroscopic performance metrics under fixed exposure conditions, while mechanistic understanding at the pore and microstructural level remains incomplete [21,22,23]. Recent review studies have consistently emphasized that reliance on macroscopic experimental observations alone is insufficient to elucidate the fundamental mechanisms of sulfate attack. It is therefore necessary to integrate experimental investigations with numerical simulations, in which ionic transport, reactive mineral formation, and the evolution of mechanical damage are explicitly coupled to enable a multiscale mechanistic understanding [24,25,26]. Within this framework, numerical models based on reactive transport theory and damage mechanics have increasingly emerged as powerful tools for investigating sulfate ingress, phase transformation processes, and the associated degradation of cement-based materials. Nevertheless, although existing models are capable of capturing, to some extent, the interactions between diffusion–reaction processes, volumetric expansion, and damage evolution, significant limitations remain in the quantitative representation of temperature effects and the evolution of pore structure during sulfate attack [27,28,29].

In this study, sulfate exposure experiments and numerical simulations are conducted to investigate cement pastes with different *w*/*c* values at different temperatures. A coupled reactive transport model is developed to represent how hydration at different *w*/*c* values affects both sulfate diffusivity and the availability of reactive aluminate hydrates. The hydration model provides phase volume fractions and porosity, which are used to estimate the effective diffusivity through Mori–Tanaka homogenization. The effects of temperature on diffusion and reaction kinetics are incorporated into the model. The analysis provides a basis for comparing sulfate resistance under the investigated conditions, subject to the reaction processes represented in the model.

## 2. Sulfate Ion Transport–Reaction Model

The water-to-cement ratio (*w*/*c*) and temperature are two key factors governing the coupled transport–reaction processes in cement paste. To quantitatively characterize their combined effects on sulfate attack behavior, a cement hydration model under different water-to-cement ratios was first established to calculate the volume fractions of the major hydration products and their variation with water-to-cement ratio. On this basis, following multiscale transport theory, the Mori–Tanaka (MT) homogenization approach was introduced to treat cement paste as a multiphase composite medium. The influence of temperature on ionic diffusion was further incorporated to construct an effective diffusion coefficient model for sulfate ions. Subsequently, by accounting for the chemical reactions between sulfate ions and hydration products and based on chemical reaction kinetics, a coupled transport–reaction model for sulfate ions in cement paste was developed. Numerical solutions of the proposed model were then employed to systematically analyze the migration behavior and reaction evolution of sulfate ions under different water-to-cement ratios and temperature conditions. The formulation of the model and its numerical implementation are described in detail in the following sections.

### 2.1. Cement Hydration Model

Cement hydration is a series of complex chemical reactions between cement and water, leading primarily to the formation of calcium silicate hydrate (C–S–H) gel, calcium hydroxide (CH), and hydrated calcium aluminate phases (CA). These hydration products collectively govern the mechanical performance and durability of hardened cement paste. The hydration process is generally divided into five distinct stages, namely the initial reaction period, induction period, acceleration period, deceleration period, and steady-state period, each characterized by different hydration rates and product formation mechanisms. Based on the fundamental mechanisms of cement hydration, the present study incorporates the nucleation and growth of hydration products, ion diffusion-controlled kinetics, and the progressive formation of product shells that hinder further reactions during the modeling process. A cement hydration kinetics model is thus established to quantitatively determine the volume fractions of individual hydration products.

First, the degree of hydration of clinker minerals is calculated, and the degree of hydration $\xi_{t}^{X}$ of clinker mineral *X* at time t can be solved using Equation (1):

(1) $$\xi_{t}^{X}=\xi_{t-1}^{X}+min(R_{t-1,1}^{X},R_{t-1,2}^{X},R_{t-1,3}^{X})\beta_{w/c}\lambda_{RH}\frac{A}{A_{0}}\exp[\frac{E_{a}^{X}}{R}(\frac{1}{T_{0}}-\frac{1}{T})]\Delta t$$

where $\xi_{t-1}^{X}$ is the degree of hydration of clinker mineral *X* in the previous time step, Δ*t* represents the time step, *A* is the Blaine surface area of cement (taken as 311 m^2^/kg), *A*_0_ is the reference surface area of cement (385 m^2^/kg), *E_a_^X^* is the apparent activation energy of clinker mineral *X*, *R* is the gas constant (8.314 J/mol/K), *T*_0_ is the reference temperature (293.15 K), and *T* represents the temperature.

The nucleation and growth rate ($R_{t,1}^{X}$), diffusion rate ($R_{t,2}^{X}$), and hydrate shell formation rate ($R_{t,3}^{X}$) of clinker mineral *X* (=C_3_S, C_2_S, C_3_A, C_4_AF) at time *t* can be expressed as [30]:

(2) $$R_{t,1}^{X}=\frac{K_{1}}{N_{1}}(1-\xi_{t}^{X}){\left[-\ln\left(1-\xi_{t}^{X}\right)\right]}^{1-N_{1}}$$

(3) $$R_{t,2}^{X}=\frac{K_{2}{\left(1-\xi_{t}^{X}\right)}^{\frac{2}{3}}}{1-{\left(1-\xi_{t}^{X}\right)}^{\frac{1}{3}}}$$

(4) $$R_{t,3}^{X}=K_{3}{\left(1-\xi_{t}^{X}\right)}^{N_{3}}$$

where *K*_1_, *N*_1_, *K*_2_, *K*_3_, and *N*_3_ are equation constants, and the values are shown in Table 1 [30,31].

The variable *β_w_*_/*c*_ represents a parameter associated with the water–cement ratio, and it can be formulated as Equation (5):

(5) $$\beta_{w/c}=\left\{\begin{array}{c} {\left[1+3.3336H^{X}\cdot w/c-\xi_{t-1}^{X})\right]}^{4},\;\mathrm{for}\;\xi_{t-1}^{X}>H^{X}\cdot w/c\\ 1,\mathrm{for}\;\xi_{t-1}^{X}\leq H^{X}\cdot w/c \end{array}\right.$$

where *H^X^* is a constant related to clinker minerals, as shown in Table 1. *λ_RH_* is a parameter related to relative humidity, which can be formulated as Equation (6):

(6) $$\lambda_{RH}={\left(\frac{RH-0.55}{0.45}\right)}^{4}$$

The formula for the relative humidity (RH) model is expressed as:

(7) $$\mathrm{RH}(\%)\;=\left\{\begin{array}{c} 100t^{0.07w/c-0.0435},\;\mathrm{for}\;w/c\;>\;0.4\\ 100t^{0.3754{\left(w/c\right)}^{2}-0.0375w/c-0.0625},\mathrm{for}\;w/c\;\leq \;0.4 \end{array}\right.$$

where *w*/*c* represents the water–cement ratio, and *t* represents the hydration time.

Ulm et al. [32] proposed the evolution formula of the volume fraction of each phase with the degree of hydration, including four main clinker phases, high-density C-S-H gel (HD C-S-H), low-density C-S-H gel (LD C-S-H), calcium hydroxide (CH), hydrated calcium aluminate (CA), hydrated calcium ferroaluminate (CAF), water, and pores. The relevant parameters are shown in Table 2. The volume of LD C-S-H (V_LD_) and the volume of HD C-S-H (V_HD_) can be calculated by Equations (8)–(10), respectively:

(8) $$V_{LD}=V_{CSH}^{C_{3}S}(\xi_{C_{3}S}^{*}-{\langle \xi_{C_{3}S}^{*}-\xi_{C_{3}S}\rangle }_{+})+V_{CSH}^{C_{2}S}(\xi_{C_{2}S}^{*}-{\langle \xi_{C_{2}S}^{*}-\xi_{C_{2}S}\rangle }_{+})$$

(9) $$V_{HD}=V_{CSH}^{C_{3}S}{\langle \xi_{C_{3}S}-\xi_{C_{3}S}^{*}\rangle }_{+}+V_{CSH}^{C_{2}S}{\langle \xi_{C_{2}S}-\xi_{C_{2}S}^{*}\rangle }_{+}$$

(10) $$\frac{V_{CSH}^{X}}{V_{c}^{0}}=\theta_{CSH}^{X}\cdot \frac{\rho_{X}^{*}/M_{X}}{\rho_{CSH}/M_{CSH}};\rho_{X}^{*}=\rho_{c}\frac{m_{X}}{\sum_{X}m_{X}}$$

where $V_{c}^{0}$ is the initial volume of cement, $\theta_{CSH}^{X}$ is the number of moles of C-S-H produced by consuming 1 mol of *X* (C_3_S/C_2_S), $V_{CSH}^{X}$ is the volume of C-S-H produced by the reactant *X* (C_3_S/C_2_S), $\rho_{X}^{*}$ is the apparent mass density of *X* (C_3_S/C_2_S) in the cement mass density *ρ_c_*.

The volume of CH in cement hydration products can be expressed by Equations (11) and (12):

(11) $$V_{CH}=V_{CH}^{C_{3}S}\cdot \xi_{C_{3}S}+V_{CH}^{C_{2}S}\cdot \xi_{C_{2}S}$$

(12) $$\frac{V_{CH}^{X}}{V_{c}^{0}}=\theta_{CH}^{X}\cdot \frac{\rho_{X}^{*}/M_{X}}{\rho_{CH}/M_{CH}};\rho_{X}^{*}=\rho_{c}\frac{m_{X}}{\sum_{X}m_{X}}$$

The volume of CA in cement hydration products can be expressed by Equations (13) and (14):

(13) $$V_{CA}=V_{CA}^{C_{3}A}\cdot \xi_{C_{3}A}$$

(14) $$\frac{V_{CA}^{X}}{V_{c}^{0}}=\theta_{CA}^{X}\cdot \frac{\rho_{X}^{*}/M_{X}}{\rho_{CA}/M_{CA}};\rho_{X}^{*}=\rho_{c}\frac{m_{X}}{\sum_{X}m_{X}}$$

The volume of CAF in cement hydration products can be expressed by Equations (15) and (16):

(15) $$V_{CAF}=V_{CAF}^{C_{4}AF}\cdot \xi_{C_{4}AF}$$

(16) $$\frac{V_{CAF}^{X}}{V_{c}^{0}}=\theta_{CAF}^{X}\cdot \frac{\rho_{X}^{*}/M_{X}}{\rho_{CAF}/M_{CA}};\rho_{X}^{*}=\rho_{c}\frac{m_{X}}{\sum_{X}m_{X}}$$

The volume *V_X_* of each clinker *X* (=C_3_S, C_2_S, C_3_A, C_4_AF) during the cement hydration process can be expressed by Equation (17):

(17) $$V_{X}=V_{X}^{0}(1-\xi_{X})$$

The volume of water *V_w_* is:

(18) $$V_{w}=V_{w}^{0}-\sum_{X}V_{w}^{X}\cdot \xi_{X}\geq 0$$

where $V_{w}^{0}$ is the initial volume of water, and $V_{w}^{X}$ is the volume of water consumed by the complete hydration of clinker phase *X*.

The volume of pores *V_V_* is:

(19) $$V_{V}=-\sum_{X}\Delta V_{V}^{X}\cdot \xi_{X}$$

The total volume of hardened cement paste is:

(20) $$V_{\mathrm{total}}=V_{c}^{0}\cdot (1+\frac{\rho_{c}}{\rho_{w}}\cdot \frac{w}{c})$$

where *w*/*c* is the water–cement ratio.

Substituting the calculated volumes *V*_i_ (LD C-S-H, HD C-S-H, clinker phase X, CH, water, voids) into Equation (21), the volume fraction of each phase can be obtained.

(21) $$f_{i}=\frac{V_{i}}{V_{\mathrm{total}}}$$

The porosity of hardened cement paste can be expressed as:

(22) $$V_{\varphi }=f_{V}+f_{w}$$

Finally, the volume fraction *f_A_* of aluminate hydrate is:

(23) $$f_{O}=1-f_{LD}-f_{HD}-\sum_{X}\;f_{X}-f_{CH}-f_{w}-f_{V}-f_{CA}-f_{CAF}$$

where *f_LD_*, *f_HD_*, *f_X_*, *f_CH_*, *f_CA_*, *f_CAF_*, *f_w_*, *f_V_* represent the volume fractions of LD C-S-H, HD C-S-H, clinker phase X, CH, CA, CAF, water, and voids, respectively.

### 2.2. Temperature-Dependent Sulfate Ion Diffusion Coefficients Model

**Figure 1:**
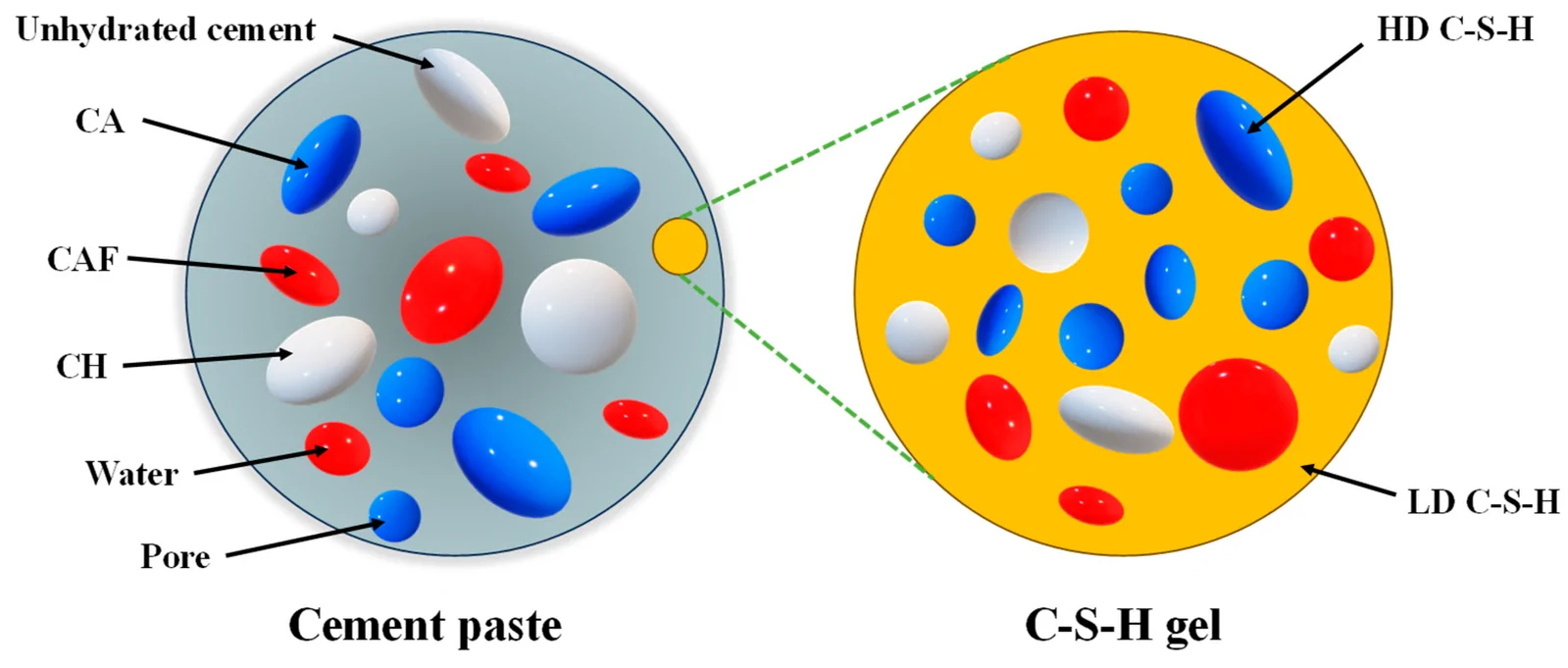
A schematic illustration of the multiscale hierarchical structure of cement paste.

This section presents a general micromechanical multi-inclusion framework for evaluating the effective diffusion coefficient of multiphase composites, with emphasis on cement-based materials. A representative volume element (RVE) Ω of a statistically homogeneous material is considered, consisting of an infinite homogeneous matrix Ω_m_ and a multi-inclusion system Ω_inc_. The matrix volume fraction and diffusion coefficient are denoted by V_m_ and D_m_, respectively. Dispersed phases with identical physical properties are treated as a single phase, such as C–S–H gel, pores and hydration products in cementitious composites. For Ω_in_c, the volume fraction and diffusion coefficient of the r-th inclusion type are denoted as Vᵣ and Dᵣ (r = 1, 2, …, *N*), where *N* is the number of inclusion types [33], as shown in Figure 1.

According to mean-field theory, the macroscopic transport behavior of porous composites can be regarded as the statistical average response of their constituent phases [34]. Following the classical work of Stratton on transport in multiphase media, a second-order concentration tensor *A_r_* is introduced to relate the average concentration gradient within the r-th phase to the applied uniform macroscopic gradient. This tensor provides an effective link between phase-scale transport properties and macroscopic boundary conditions, enabling multiscale coupling from microstructure to macroscopic behavior. Combined with the porous media transport model proposed by Xu et al. [33], the effective diffusion behavior of porous composites is obtained by a weighted average of phase contributions. Accordingly, the overall effective diffusion coefficient depends on the intrinsic diffusion coefficients, volume fractions, and corresponding concentration tensors of all phases, allowing a realistic description of ion transport in multiphase systems without explicitly resolving complex microstructural details. Based on the MT method, the diffusion coefficient of the porous medium can be expressed as Equations (24)–(26).

(24) $$\langle D_{eff}^{MT}\rangle =D_{m}+\left[\sum_{r=1}^{N}\left(D_{r}-D_{m}\right)V_{r}\cdot \langle A_{r}\rangle \right]{\left[V_{m}+\sum_{r=1}^{N}V_{r}\langle A_{r}\rangle \right]}^{-1}$$

(25) $$\langle A_{r}\rangle =\frac{1}{3}\left(\frac{2D_{m}}{\left(1-Q_{r}\right)D_{m}+Q_{r}D_{r}}+\frac{D_{m}}{2Q_{r}D_{m}+\left(1-2Q_{r}\right)D_{r}}\right)$$

(26) $$Q_{p}=\left\{\begin{array}{cc} \frac{1}{2}\left\{1+\frac{1}{k_{p}^{2}-1}\left[1-\frac{k_{p}}{2\sqrt{k_{p}^{2}-1}}\ln\left(\frac{k_{p}+\sqrt{k_{p}^{2}-1}}{k_{p}-\sqrt{k_{p}^{2}-1}}\right)\right]\right\}, & k_{p}>1\\ \frac{1}{2}\left\{1+\frac{1}{k_{p}^{2}-1}\left[1-\frac{k_{p}}{\sqrt{1-k_{p}^{2}}}\arctan\left(\frac{\sqrt{1-k_{p}^{2}}}{k_{p}}\right)\right]\right\}, & k_{p}<1 \end{array}\right.$$

where *κ_p_* denotes the aspect ratio of spheroidal inclusions. When *κ_p_* > 1, the inclusions are prolate spheroids, whereas *κ_p_* < 1 corresponds to oblate spheroids. For special cases, *Q_p_* = 1/3 for spherical inclusions κ = 1, and *Q_p_* = 0 for platelet-shaped inclusions *κ* = 0.

Based on multiscale theory, sulfate ion transport in cement-based materials can be described by a hierarchical homogenization approach, in which effective diffusion coefficients are evaluated successively from the microscale to the macroscale. In this framework, the transport properties of C-S-H gel are first homogenized at the submicron scale and then introduced as an equivalent phase in the diffusion analysis at the cement paste scale, enabling continuous coupling across scales. At the submicron level, C-S-H gel is commonly modeled as an inclusion–matrix–composite composed of HD C-S-H and LD C-S-H [35]. The dense HD C-S-H, with small pore sizes, strongly hinders ion transport, whereas the more porous and better-connected LD C-S-H provides the primary diffusion pathways. Consequently, their spatial arrangement governs the overall diffusion behavior of the C-S-H gel. Accordingly, the effective diffusion coefficient of the C-S-H gel can be expressed as:

(27) $$\langle D_{CSH}\rangle =D_{CSHL}+\frac{V_{CSHH}\left(D_{CSHH}-D_{CSHL}\right)\cdot \langle A_{CSHH}\rangle }{V_{CSHL}+\langle A_{CSHH}\rangle V_{CSHH}}$$

(28) $$V_{CSHH}=\frac{V_{CSHH}}{V_{CSHH}+V_{CSHL}}$$

(29) $$A_{CSHH}=\frac{1}{3}\left(\frac{2D_{CSHL}}{\left(1-Q_{c}\right)D_{CSHL}+Q_{c}D_{CSHH}}+\frac{D_{CSHL}}{2Q_{c}D_{CSHL}+\left(1-2Q_{c}\right)D_{CSHH}}\right)$$

where the LD C-S-H diffusion coefficient D_CSHL_ = 8.2 × 10^−12^ m^2^/s, the HD C-S-H diffusion coefficient D_CSHH_ = 2 × 10^−13^ m^2^/s [36,37], and the shape factor is Q_c_ = 1/3.

At the microscale, cement paste can be regarded as a complex multiphase system composed of various hydration products and unhydrated particles, including C-S-H gel, unhydrated cement particles (C_3_S, C_2_S, C_3_A, C_4_AF), CH, CA, CAF, pores, and aqueous solution phases. For multiscale modeling and the calculation of effective diffusion coefficients, the C-S-H gel is treated as the matrix phase, while the remaining phases and pores are considered inclusions, forming a typical inclusion–matrix–composite structure. The volume fractions of the matrix phase and the pore inclusion phase are given using Equations (30) and (31), respectively.

(30) $$V_{m}=f_{LCSH}+f_{HCSH}$$

(31) $$V_{p}=f_{CH}+f_{CA}+f_{CAF}+f_{u}+f_{o}$$

Accordingly, the overall effective diffusion coefficient of the cement paste can be expressed as:

(32) $$\langle D_{CP}\rangle =D_{CSH}+\frac{\left[V_{p}\left(D_{p}-D_{CSH}\right)\cdot \langle A_{p}\rangle +V_{\varphi }\left(D_{\varphi }-D_{CSH}\right)\cdot \langle A_{\varphi }\rangle \right]}{\left[V_{CSH}+\langle A_{p}\rangle V_{p}+\langle A_{\varphi }\rangle V_{\varphi }\right]}$$

(33) $$A_{p}=\frac{1}{3}\left(\frac{2D_{CSH}}{\left(1-Q_{p}\right)D_{CSH}+Q_{p}D_{p}}+\frac{D_{CSH}}{2Q_{p}D_{CSH}+\left(1-2Q_{p}\right)D_{p}}\right)$$

(34) $$A_{\varphi }=\frac{1}{3}\left(\frac{2D_{CSH}}{\left(1-Q_{\varphi }\right)D_{CSH}+Q_{\varphi }D_{\varphi }}+\frac{D_{CSH}}{2Q_{\varphi }D_{CSH}+\left(1-2Q_{\varphi }\right)D_{\varphi }}\right)$$

where the diffusion coefficients of unhydrated cement particles (C_3_S, C_2_S, C_3_A, and C_4_AF), CH, CA, and CAF are all assumed to be zero (*D_p_* = 0).

The diffusion process of ions in solution exhibits a pronounced dependence on temperature, primarily because temperature variations directly affect the intensity of ionic thermal motion and the mobility of ions in the solvent. As temperature increases, the average kinetic energy of ions rises, leading to more vigorous Brownian motion under thermal fluctuations and thereby increasing the probability of ions traversing a given spatial distance within a unit time. Concurrently, elevated temperature is generally associated with a reduction in solution viscosity, which decreases the resistance encountered by ions during migration among solvent molecules, further enhancing ionic mobility and diffusion flux. In contrast, at lower temperatures, ionic thermal motion is restricted and solution viscosity is higher, resulting in a marked suppression of the diffusion process. From thermodynamic and kinetic perspectives, diffusion is inherently a non-equilibrium transport process driven by concentration gradients, and temperature governs the diffusion rate by modulating ionic mobility and diffusion coefficients. Therefore, when describing the transport behavior of solutes such as sulfate ions in solution, the influence of temperature must be explicitly considered to ensure that the model accurately captures the diffusion characteristics under different environmental temperature conditions. According to the Nernst–Einstein equation, the diffusion coefficient of sulfate ions in pure water can be expressed as [38]:

(35) $$D_{\varphi }=\frac{RT}{F^{2}}\frac{\lambda }{\left|z\right|}$$

where *T* is the thermodynamic temperature, *F* is the Faraday constant, λ is the molar ionic conductivity (S·m^2^·mol^−1^), and z is the charge number of the sulfate ion.

### 2.3. Transport–Reaction Model and Numerical Solution

The sulfate ions from the external environment penetrate cementitious materials and react with hydrated calcium aluminate phases to form ettringite (AFt). Following the simplified approach adopted by Ikumi et al., all hydrated aluminate phases CA participating in this reaction are represented by calcium monosulfoaluminate hydrate ($C_{4}A\overset{-}{S}H_{12}$) in the present model [39]. The conversion of this phase to AFt increases the solid volume. When crystal growth is constrained by the surrounding matrix, crystallization pressure can develop and promote the initiation and propagation of microcracks. This process contributes to expansion, microstructural deterioration, and mechanical degradation under sulfate exposure. The corresponding chemical reaction can be expressed as follows [40]:

(36) $$2{\mathrm{Ca}}^{2+}+2{\mathrm{SO}}_{4}^{2-}+C_{4}A\bar{S}H_{12}+20H_{2}O\to C_{6}A{\bar{S}}_{3}H_{32}$$

The influence of temperature on chemical reaction rates can be quantitatively described using the empirical model proposed by Santhanam et al. [41]. This model is based on the Arrhenius equation and is employed to characterize the effect of temperature on reaction kinetics. The model is expressed as:

(37) $$H_{k}=\exp\left(-\frac{E_{a}}{R}\left(\frac{1}{T}-\frac{1}{T_{ref}}\right)\right)$$

where *E_a_* represents the apparent activation energy term in the Arrhenius formulation. In this study, *E_a_*/R was taken as 4430.7 K. *T_ref_* denotes the reference temperature, which is the baseline temperature adopted in the model, with a value of 298 K.

Assuming that sulfate ions are transported from the specimen surface into the interior along the x-direction, the diffusion process can be regarded as a one-dimensional transport problem. The coordinate x is defined from the interior boundary (x = 0) toward the exposed surface (x = L). Accordingly, the governing diffusion model for sulfate ions can be expressed as:

(38) $$\frac{𝜕C_{S}}{𝜕t}=\frac{𝜕}{𝜕x}\left(D_{cp}\frac{𝜕C_{S}}{𝜕x}\right)+JC_{S}$$

(39) $$J=-H_{k}k_{v}C_{\mathrm{CA}}$$

The formation concentration of ettringite is expressed as:

(40) $$C_{AFt}=0.5\int_{0}^{t}H_{k}k_{v}C_{AFm}C_{S}dt$$

(41) $$C_{AFm}=C_{AFm0}-C_{AFt}$$

where *k_v_* is the reaction rate constant between sulfate ions and hydrated calcium aluminate in cementitious materials, *k_v_* = 1.22 × 10^−9^ m^3^/(mol·s) [40], and *C_AFm_* is the concentration of calcium monosulfoaluminate hydrate in the cement paste (mol/m^3^). *C_AFm_*_0_ represents the initial AFm concentration; its mass fraction is determined in Section 2.1 and then converted to molar concentration via molar mass.

The initial and boundary conditions are given as:

(42) $$\left\{\begin{array}{cc} \mathrm{Initial}: & C_{S}(x,\;0)=0\\ \mathrm{Boundary}: & C_{S}(L,\;t)=C_{S0};{\left.\frac{𝜕C_{S}}{𝜕x}\right|}_{x=0}=0 \end{array}\right.$$

where *C_S_*_0_ is the prescribed free-sulfate concentration at the exposed surface x = L. The boundary at x = 0 represents the epoxy-sealed rear surface and is therefore assigned a zero-flux condition. The penetration depth measured from the exposed surface is defined as d = L − x.

Specifically, the original diffusion–reaction equation is subject to nonhomogeneous boundary conditions, which makes the direct solution of the nonlinear partial differential equation challenging. The transformation in Equation (43) homogenizes the prescribed concentration boundary at x = L. This transformation significantly reduces the complexity of discretization and numerical iteration, enabling a more efficient computation of the sulfate ion concentration distribution.

(43) $$u=C_{S}-\frac{xC_{S0}}{L}$$

The transformed diffusion model and the corresponding boundary conditions can be expressed as [42]:

(44) 𝜕u𝜕t=𝜕𝜕xDcp𝜕u𝜕x+Ju+xLCS0,x∈[0,L]Boundary condition: 𝜕u𝜕x(0,t)=−CS0L, u(L,t)=0 Initial condition: u(x, 0)=−xCS0L0≤x<L

Before the numerical solution of Equation (38), the continuous variables are discretized by dividing the solution domain into uniformly spaced grids. With a time step of Δ*t*, the total exposure time of concrete in the sulfate solution is divided into *K* intervals, and an arbitrary time during sulfate attack can be expressed as:

(45) $$t^{k}=(k-1)\Delta t,\;k=0,1,\ldots ,K$$

To improve the accuracy and numerical stability of the partial differential equation, the Crank–Nicolson scheme is employed to discretize Equation (38) in time, with first-order central differences used for spatial approximation. Accordingly, the finite-difference governing equation of the sulfate ion diffusion–reaction model can be obtained as:

(46) $$u^{k}=A^{-1}\left(Bu^{k-1}-F\right)$$

where the matrices **A**, **B** and the vector **F** are defined as:

(47) $$A=\frac{1}{\Delta t}a^{T}+\frac{1}{2}{\left(Ja+b\right)}^{T}$$

(48) $$B=\frac{1}{\Delta t}a^{T}-\frac{1}{2}{\left(Ja+b\right)}^{T}$$

(49) $$F=\frac{\Delta t\Delta xJC_{S0}}{4M}\left\{\begin{array}{c} 0\\ 2(2-1)\\ \vdots \\ 2(M-1)\\ M \end{array}\right\}$$

where the matrix **a** is the mass matrix, representing the contribution of each spatially discretized element to the temporal rate of change of u. The matrix **b** is the diffusion stiffness matrix, describing the diffusion transport characteristics of sulfate ions within the concrete.

(50) $$a=\frac{\Delta x}{6}\left[\begin{array}{ccccc} 2 & 1 & \ldots & 0 & 0\\ 1 & 4 & \ldots & 0 & 0\\ \vdots & \vdots & \ddots & \vdots & \vdots \\ 0 & 0 & \ldots & 4 & 1\\ 0 & 0 & \ldots & 1 & 2 \end{array}\right]$$

(51) $$b=\frac{D}{\Delta x}\left[\begin{array}{ccccc} 1 & -1 & \ldots & 0 & 0\\ -1 & 2 & \ldots & 0 & 0\\ \vdots & \vdots & \ddots & \vdots & \vdots \\ 0 & 0 & \ldots & 2 & -1\\ 0 & 0 & \ldots & -1 & 1 \end{array}\right]$$

By performing iterative calculations, *u^k^* at any corrosion time *t_k_* can be obtained. Finally, using the transformation relationship in Equation (52), the spatiotemporal distribution of sulfate ion concentration *C_S_* within the cement paste can be derived.

(52) $$C_{S}=u^{k}+\frac{xC_{S0}}{L}$$

The coupled transport–reaction model was implemented as a numerical program in MATLAB R2024a. MATLAB was selected for its efficient numerical-computing environment and strong data-processing and visualization capabilities, which facilitate the solution of the coupled governing equations and the direct generation of graphical outputs. The developed program calculates and exports the temporal and spatial distributions of the model variables at the prescribed numerical precision. The resulting numerical predictions and their comparison with the experimental data are presented and discussed in detail in Section 4.

## 3. Experimental Program

### 3.1. Sulfate Ion Attack Test

Reference Portland cement and deionized water were used to prepare cement pastes with water-to-cement ratios of 0.3, 0.4, and 0.5. These ratios were selected as controlled low, intermediate, and high levels to investigate the influence of capillary porosity. The fresh pastes were cast into 20 mm × 20 mm × 50 mm prisms and cured under standard conditions for 28 days. Before sulfate exposure, five of the six surfaces of each specimen were sealed with epoxy resin, leaving only one surface with a dimension of 20 mm × 20 mm exposed to the sulfate solution. This configuration established a nominally one-dimensional sulfate ingress condition. The chemical composition of the cement is provided in Table 3.

**Figure 2:**
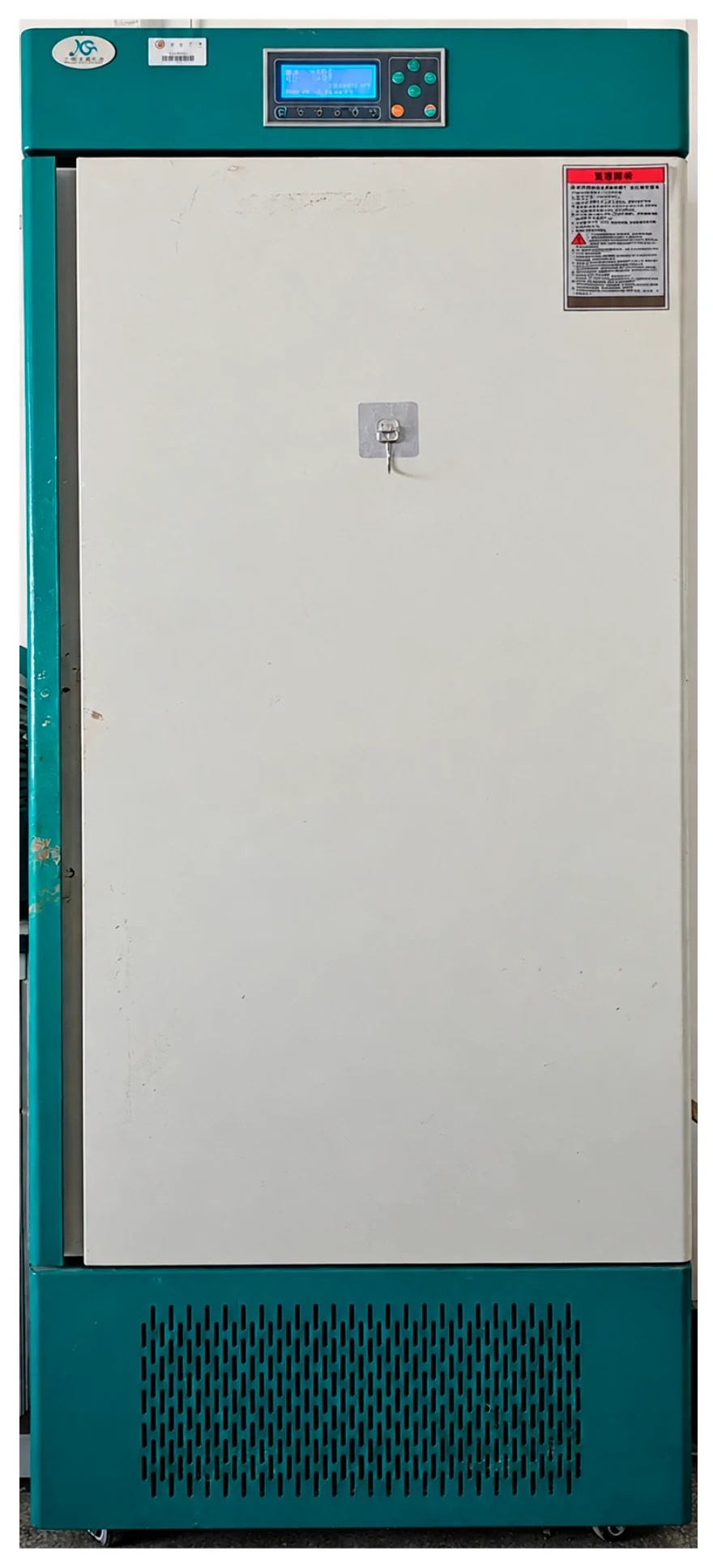
Temperature-controlled chamber.

Analytical-reagent-grade anhydrous sodium sulfate was supplied by Sinopharm Chemical Reagent Co., Ltd.,Shanghai, China. According to the manufacturer’s specification, the sodium sulfate content was not less than 99.0%. The reagent was dissolved in deionized water to prepare a sodium sulfate solution with a mass fraction of 5.0%. The solution pH was maintained within the neutral range to ensure that the sulfate medium remained free from interference by other ions. Cement paste specimens were placed in sealed plastic containers with the solution, maintaining a specimen-to-solution volume ratio of 1:50 to ensure a relatively stable sulfate ion concentration. The sealed containers were then placed in a temperature-controlled chamber, and the temperature was adjusted and maintained at the target experimental value, as illustrated in Figure 2. Sulfate exposure tests were conducted under different temperature conditions according to the experimental scheme summarized in Table 4. At each measurement time, three replicate specimens were tested for each experimental condition. Group 1 was designed to validate the accuracy of the proposed model, while Experimental Groups 2 and 3 were designed to systematically investigate the effects of temperature and water-to-cement ratio on the transport–reaction behavior of sulfate ions.

### 3.2. Quantitative Characterization

**Figure 3:**
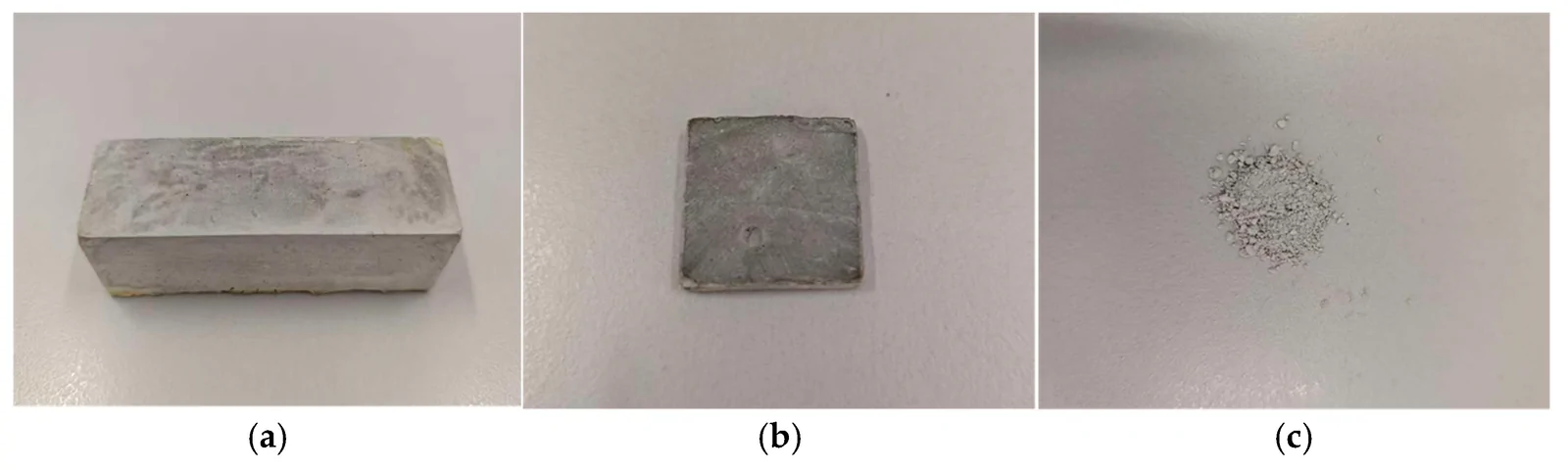
Sample handling process. (**a**) Samples; (**b**) Slices; (**c**) Powder.

On the testing date, the specimens were sectioned along the direction of sulfate attack at 5 mm intervals into 2 mm-thick slices, which were then immersed in isopropanol for 7 days to stop further hydration. The slices were subsequently dried in a vacuum oven at 40 °C for 3 days to remove residual isopropanol. The dried slices were ground into powder, sieved through a 180-mesh screen, and the mass of the sieved powder *m_p_* was recorded. All these procedures were carried out in a nitrogen-controlled glove box to prevent carbonation, as shown in Figure 3.

The total sulfate content at different depths was determined by gravimetric BaSO_4_ precipitation after acid digestion of the powdered samples. The mass of precipitated BaSO_4_ (*m_s_*) was used to calculate the total sulfate mass fraction *w_ST_*, according to Equation (53):

(53) $$w_{ST}=\frac{0.412m_{S}}{0.5m_{p}}$$

The coefficient 0.412 represents the mass ratio of sulfate ion to BaSO_4_. Because the samples were subjected to acid digestion, the measured value included free sulfate and sulfate bound in solid phases. Therefore, the gravimetric result represented the total sulfate mass fraction and could not be compared directly with the mobile aqueous sulfate represented by *C_S_* in Equation (38). In the simplified reaction system adopted in this study, chemically bound sulfate in crystalline phases formed during the erosion process is attributed to ettringite. Based on quantitative characterization, the calculated mass fraction of AFt is *w*_AFt_. Under simplified assumptions, the mass fraction of sulfate contained in AFt is calculated as:

(54) $$w_{S\mathrm{AFt}}=\frac{3M_{{\mathrm{SO}}_{4}}}{M_{\mathrm{AFt}}}w_{\mathrm{AFt}}$$

where the molar masses of sulfate and ettringite are M_SO4_ = 96.06 g·mol^−1^ and M_AFt_ = 1255 g·mol^−1^.

After subtracting the sulfate incorporated into AFt, the free sulfate mass fraction *w_FS_* was reconstructed as

(55) $$w_{FS}=w_{ST}-w_{S\mathrm{AFt}}$$

The corrosion products were quantitatively characterized using the XRD–Rietveld method, with corundum (α-Al_2_O_3_) as the internal standard at a content of 20%. Phase composition analysis was conducted via quantitative X-ray diffraction (QXRD) using a D8 ADVANCE diffractometer (Bruker, Karlsruhe, Germany). During the measurement, the scanning range was set from 5° to 65° 2θ with a step size of 0.02°, providing quantitative information on the phase composition of the samples [43].

The slices were vacuum-dried at 40 °C to remove residual isopropanol while avoiding the more pronounced alteration of the hydrate assemblage that may occur during conventional oven drying at 60–105 °C. Previous studies on ettringite-rich cementitious materials have shown that the reduction in ettringite crystallinity caused by drying at 40 °C is less pronounced than that caused by drying at 60 or 105 °C [44]. Therefore, 40 °C was selected as a compromise between effective solvent removal and preservation of the hydrate assemblage, rather than being regarded as an artifact-free drying temperature. Nevertheless, ettringite stability is governed jointly by temperature, water-vapor activity, and treatment duration; therefore, 35–40 °C cannot be considered a universally applicable decomposition threshold. Under vacuum conditions, the reduced water-vapor pressure may promote partial dehydration of ettringite even at relatively low temperatures [45,46]. Consequently, vacuum drying at 40 °C for 3 days may reduce the intensity of the AFt diffraction peaks, leading to systematic underestimation of the crystalline AFt content determined by Rietveld refinement. Because 20 wt.% corundum was used as an internal standard, part of the material associated with the loss of AFt crystallinity may be reflected as an increase in the calculated amorphous content. Overall, this protocol represents a practical compromise that provides adequate removal of residual solvent and evaporable water while minimizing, as far as practicable, the dehydration of ettringite.

## 4. Results and Discussion

### 4.1. Model Validation

**Figure 4:**
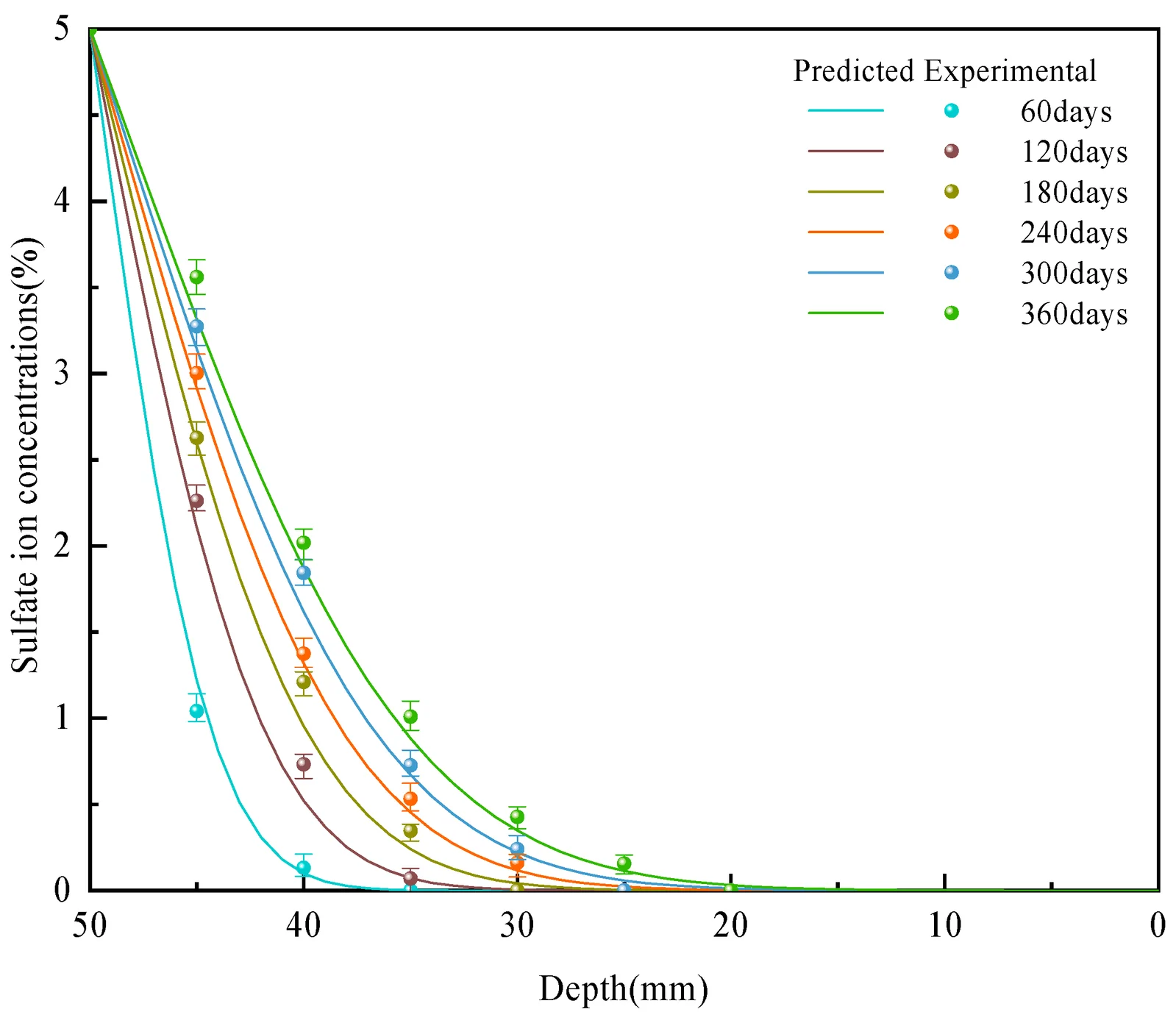
Concentration of sulfate ions obtained by the established model and the experiments of depth at different immersion times.

Figure 4 compares the sulfate ion concentration profiles predicted by the coupled transport and reaction model with measurements obtained at six immersion durations. The simulated results are obtained by solving Equation (43), while the experimental results are corrected via Equation (55). The simulations reproduced the decrease in sulfate concentration with increasing distance from the exposed surface and its progressive increase with immersion time, thereby capturing the principal spatial and temporal characteristics of sulfate transport. After excluding the prescribed 5% surface boundary values at x = 50 mm and the experimental zero-concentration points, 21 paired observations were retained for validation. “Depth” is measured from the sealed rear face, with the exposed surface located at Depth = 50 mm. The experimental values represent the means of three replicate specimens for each experimental condition, and the error bars indicate ±1 standard deviation (SD). The comparison yielded R^2^ = 0.985 and a pooled mean absolute relative error of 13.64%. Despite this overall agreement, the model underestimates concentrations at several locations at exposure times of 120 days and beyond. At some locations, the predicted curves fall below the lower limits of the experimental error bars, indicating discrepancies beyond the displayed experimental variability. Relative deviations are particularly sensitive near the penetration front, where low concentrations amplify the percentage errors associated with small absolute differences. One possible contributor to the underestimation is sulfate-induced microcracking, which may create additional transport pathways and enhance sulfate ingress. This feedback is not explicitly represented in the present model. The limited spatial resolution of the measurements may also contribute to local discrepancies. Overall, the model captures the main spatial and temporal trends, but its predictions of local concentrations at later exposure times require further refinement.

Overall, the sulfate ion concentration decreases gradually with increasing penetration depth, reflecting diffusion-driven transport from the exposed surface toward the interior of the cement paste. With increasing immersion time, sulfate ions continuously penetrate deeper into the material, and the effective penetration depth progressively expands, exhibiting typical time-dependent diffusion-controlled behavior [5]. Previous experimental studies on external sulfate attack have consistently reported similar depth-dependent sulfate concentration profiles, confirming that sulfate ingress is governed by coupled diffusion and chemical reaction processes [17]. These observations indicate that the proposed model can effectively capture the diffusion–reaction behavior of sulfate ions in cement paste and provides a solid theoretical basis for further investigation of the effects of temperature and water-to-cement ratio on sulfate transport.

**Figure 5:**
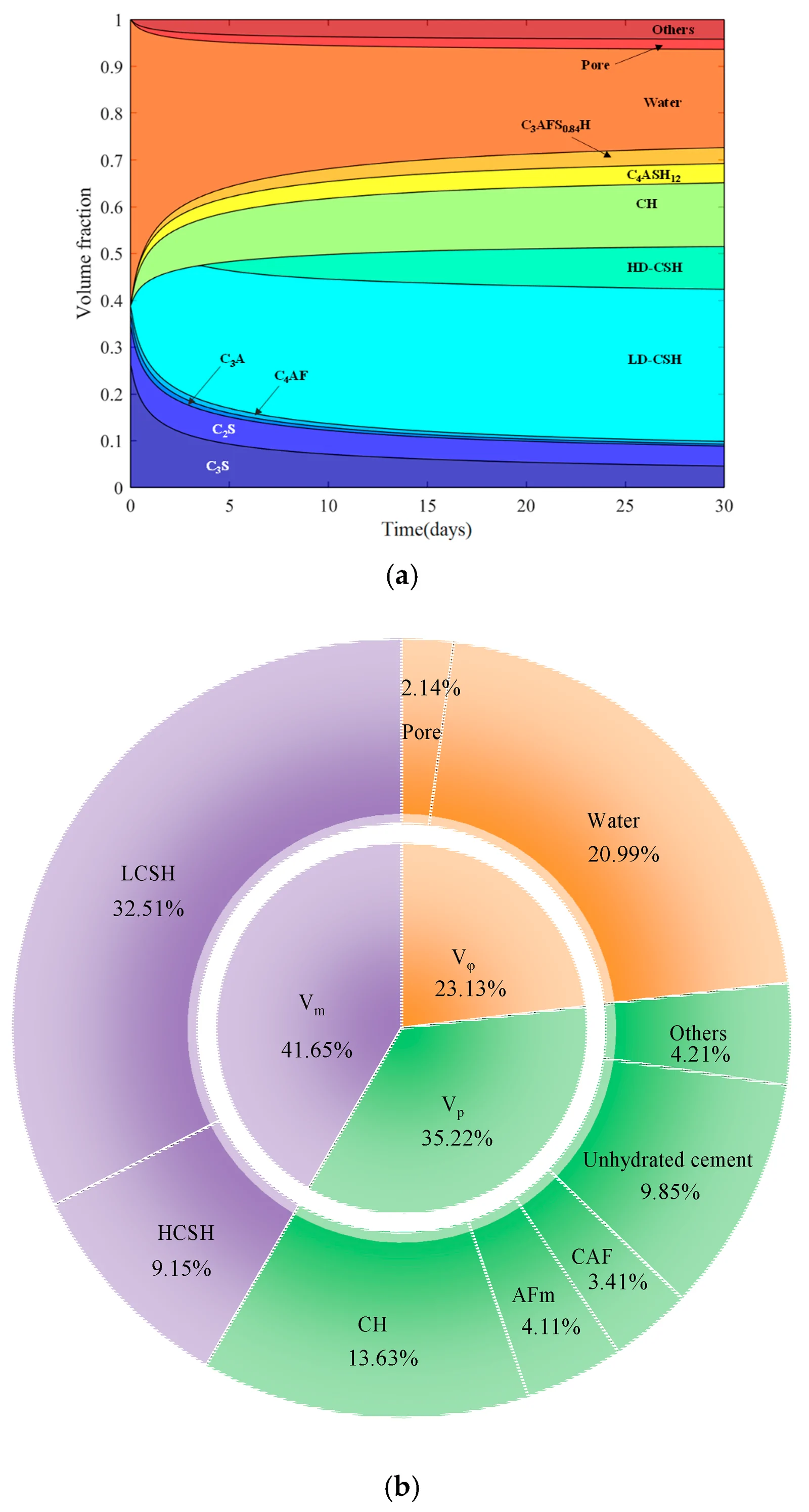
Hydration products volume fractions by the established model at *w*/*c* = 0.5: (**a**) Evolution of phase volume fractions in cement paste over hydration time; (**b**) Phase volume fractions at 28 days.

Figure 5a illustrates the evolution of the volume fractions of various hydration products in cement paste with a water-to-cement ratio of 0.5 during hydration. In the early stage, rapid reactions of cement clinker minerals lead to the continuous formation of major hydration products, such as C-S-H gel and CH, resulting in a pronounced increase in their volume fractions [47,48]. Meanwhile, capillary pores are progressively filled by hydration products, causing a continuous reduction in porosity and indicating a transition of the microstructure from a loose to a denser state [49]. As hydration proceeds, the variations in the volume fractions of different hydration products gradually level off after approximately 30 days, and the hydration system approaches a quasi-steady state. This overall evolution shows good agreement with the predictions of the classical Powers hydration model, thereby validating the rationality of the hydration calculation approach adopted in this study [49,50].

Figure 5b presents the volume fraction distribution of various hydration products at a hydration age of 30 days. Based on the previously established multiscale effective diffusion coefficient model, the calculated volume fractions of each hydrated phase were substituted into the diffusion model in Equation (32) to obtain the overall effective diffusion coefficient of sulfate ions in cement paste. The results indicate that, at this hydration age, the effective diffusion coefficient of sulfate ions is 6.83 × 10^−12^ m^2^/s. This value reflects the combined influence of the multiphase microstructure on sulfate ion transport behavior.

**Figure 6:**
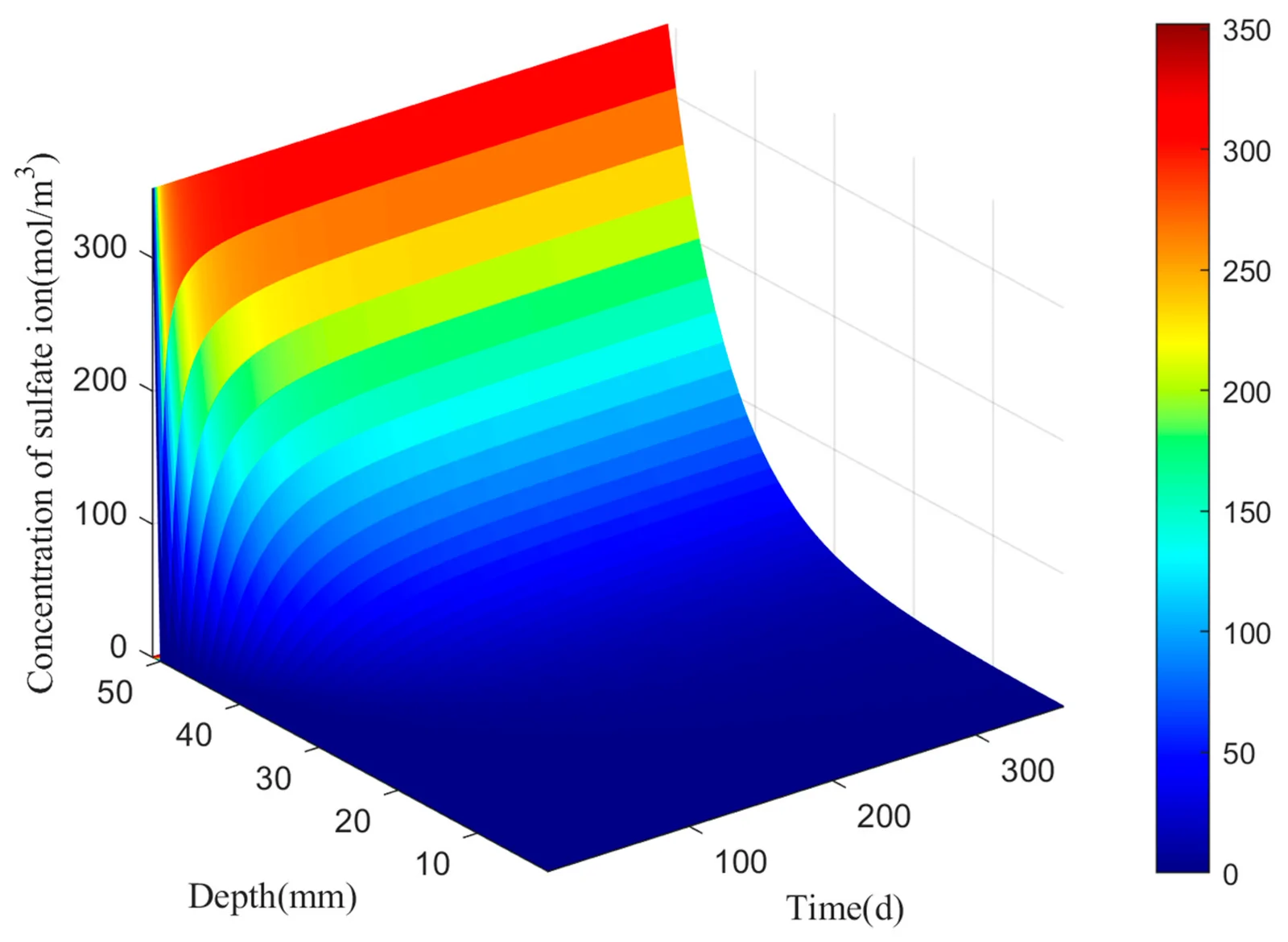
Concentration distribution of sulfate ions.

Furthermore, the effective diffusion coefficient obtained above was incorporated into the sulfate ion transport–reaction model in Equation (44) to numerically simulate the spatiotemporal evolution of sulfate ions during sulfate attack, as shown in Figure 6. The results indicate that, with increasing exposure time, sulfate ions continuously migrate from the material surface toward the interior, and the affected region progressively expands, exhibiting pronounced time-dependent diffusion behavior. In addition, the temporal evolution of sulfate ion concentration is distinctly nonlinear rather than linearly increasing. At the early stage of attack, sulfate ions are mainly concentrated near the surface, where the concentration rises rapidly. As the attack progresses, ions gradually diffuse into deeper regions, and their transport is jointly governed by diffusion resistance and chemical reaction consumption, leading to a change in the rate of concentration evolution. These results demonstrate that sulfate ion transport is inherently a nonlinear process controlled by the coupling of diffusion and reaction mechanisms.

**Figure 7:**
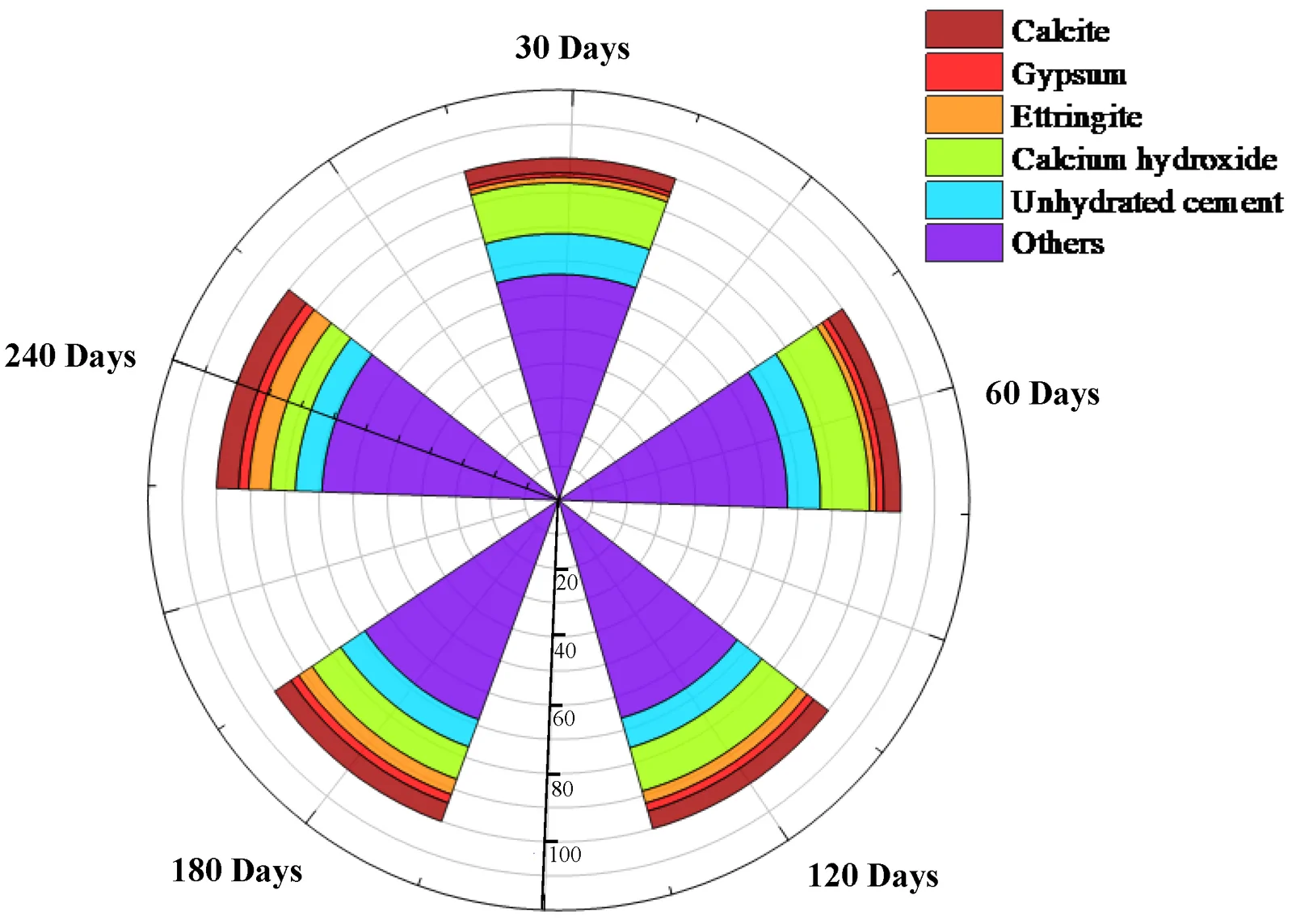
Quantitative characterization results at a depth of 5 mm.

The quantitative characterization results of hydration products at a depth of 5 mm are shown in Figure 7. As sulfate ions continuously penetrate into the cement paste, chemical reactions within the system proceed, leading to a pronounced increase in AFt formation with increasing exposure time. At the early stage of sulfate attack, sulfate ions mainly react with CH, which serves as a calcium source for AFt formation, resulting in a gradual decrease in the volume fraction of CH [51]. With further exposure, sulfate ions diffuse into deeper regions, and AFt continues to form, accompanied by a continuous increase in its volume fraction, indicating that sulfate attack progressively develops from the surface toward the interior. During this process, unhydrated cement particles undergo further hydration or participate in secondary reactions under the sustained aggressive environment, leading to a gradual reduction in their volume fraction over time.

When sulfate ions diffuse into concrete, they first react with monosulfate (AFm) to form ettringite, while gypsum is mainly generated after AFm is depleted through reactions involving calcium ions released from calcium hydroxide. As shown in Figure 7, the gypsum content remains relatively low throughout the exposure period, reaching only about 3.1 wt.% at 240 days, which is markedly lower than the ettringite content. Therefore, gypsum formation is neglected in the model to simplify the calculations without significantly affecting the overall description of sulfate attack.

### 4.2. Effect of Temperature

**Figure 8:**
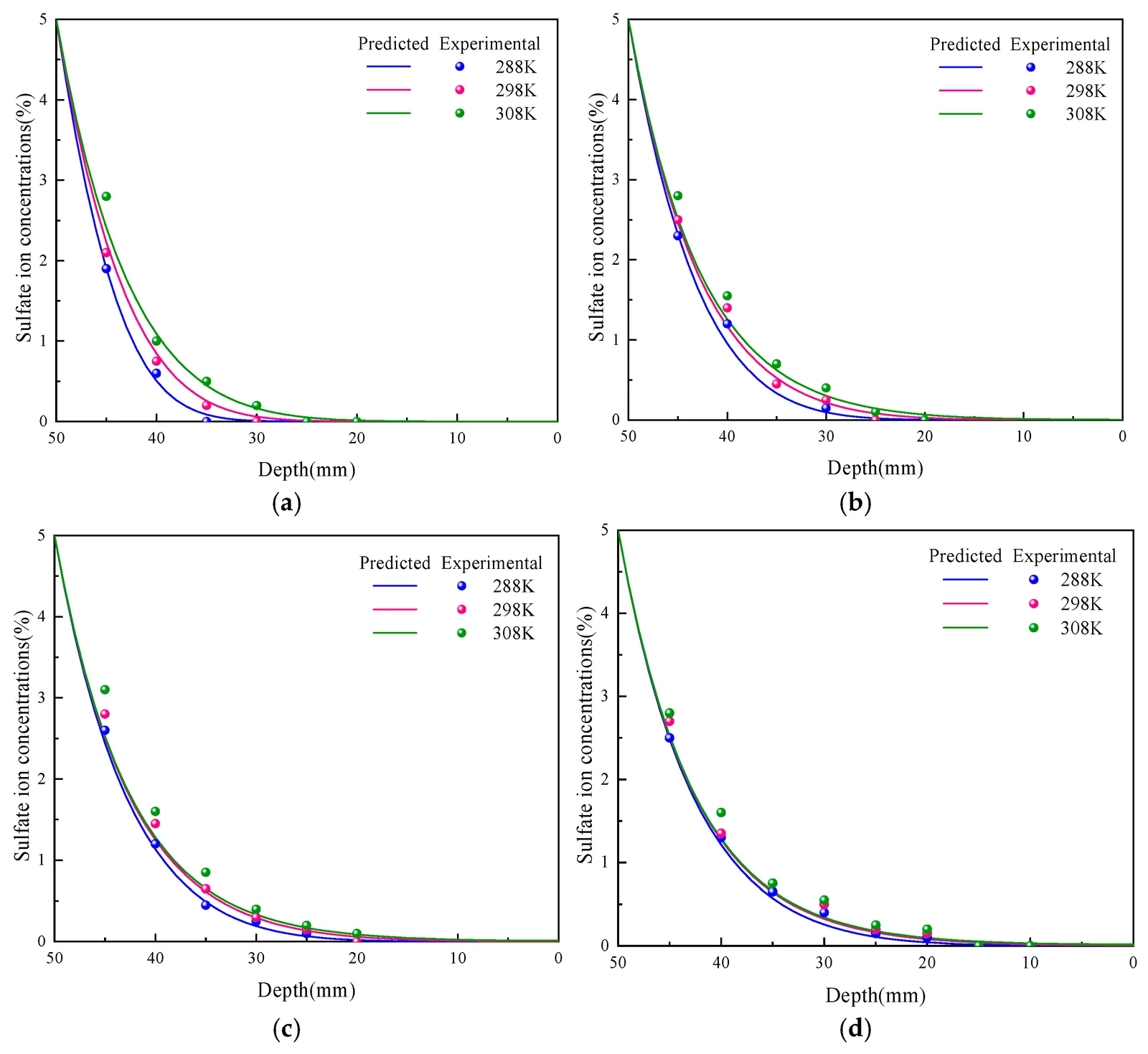
The concentration of sulfate ions at different temperatures: (**a**) 60 days; (**b**) 120 days; (**c**) 180 days (**d**) 240 days.

Figure 8 compares the simulated and measured sulfate ion concentrations in cement paste exposed to a 5% sulfate solution at three temperatures (288 K, 298 K, and 308 K). The three temperatures were chosen as evenly spaced cool, ambient, and warm exposure conditions to separate temperature sensitivity over a practical laboratory range. The experimental values represent the mean of three replicate measurements for each temperature. The results show that, at all temperatures, the simulated concentration profiles are consistent with the experimental trends, indicating that the developed model can predict the distribution of sulfate ions in cement paste under different temperature conditions with acceptable accuracy. For all temperatures considered, the discrepancies between simulated and measured sulfate ion concentrations are within 15%. Taking the case at an immersion time of 120 days as an example, the experimental values at a depth of 15 mm exceed the corresponding simulated results by 9.23%, 10.26%, and 12.63% at 288 K, 298 K, and 308 K, respectively. Overall, the proposed model adequately captures the influence of temperature on external sulfate attack in cement paste.

It can be clearly observed that the overall sulfate ion concentration in cement-based materials increases with increasing environmental temperature. Under identical exposure durations and external sulfate boundary conditions, the internal sulfate ion concentration is significantly higher at elevated temperatures than at lower temperatures. This behavior is mainly attributed to the simultaneous increase in the effective diffusion coefficient of sulfate ions and the associated chemical reaction rate constants with increasing temperature. According to the Arrhenius relationship, both diffusion and chemical reaction processes exhibit pronounced temperature sensitivity, and the enhanced thermal motion of ions at higher temperatures accelerates sulfate ion migration in the pore solution [52,53]. Moreover, the results indicate that, at higher temperatures, sulfate ions are able to penetrate deeper into the material within the same exposure period, as evidenced by an inward shift in the sulfate ion concentration profiles. This observation demonstrates that increasing temperature markedly enhances sulfate ion transport in cement-based materials, promoting the continuous advancement of the diffusion front.

**Figure 9:**
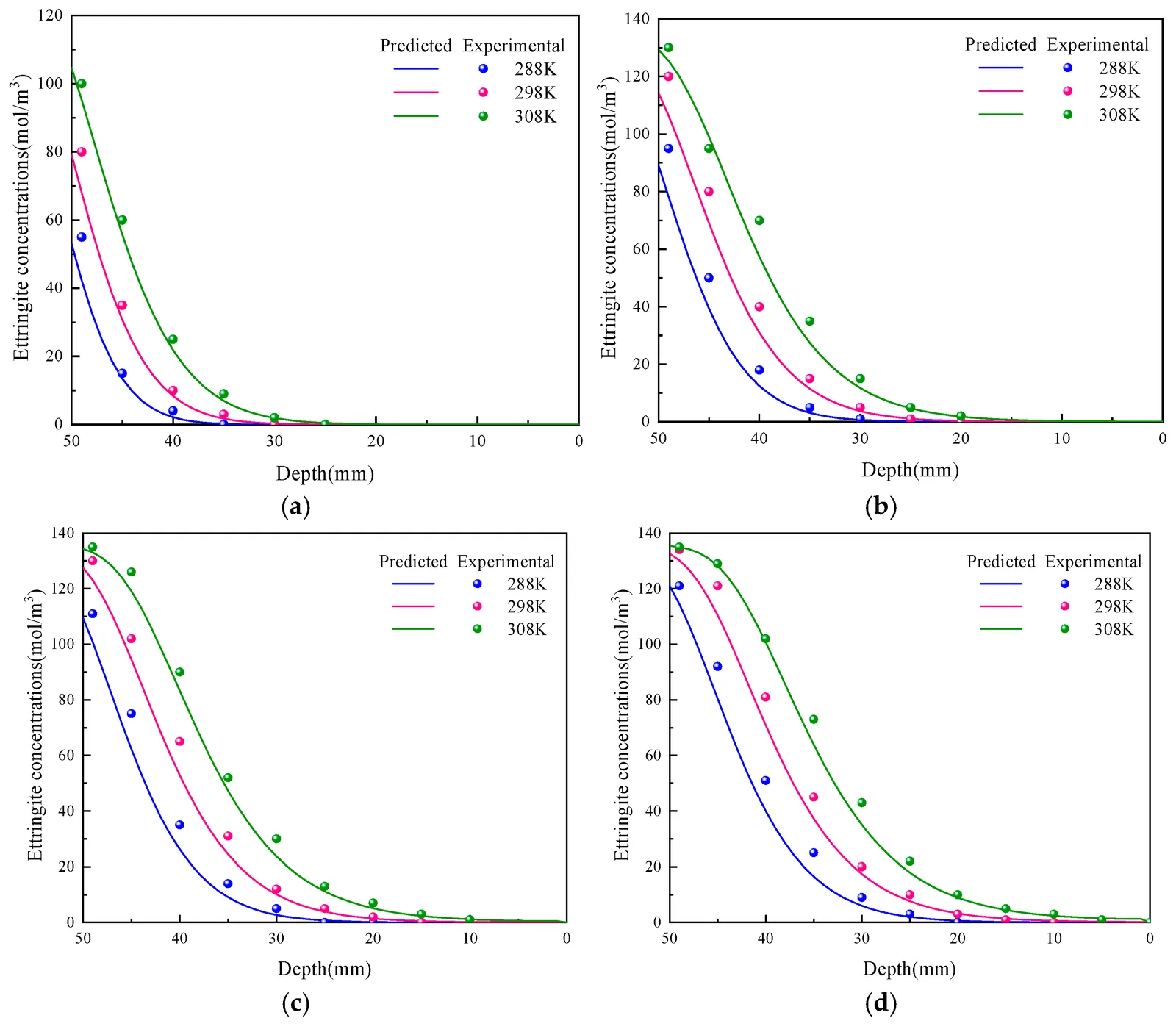
The concentration of AFt at different temperatures: (**a**) 60 days; (**b**) 120 days; (**c**) 180 days; (**d**) 240 days.

**Figure 10:**
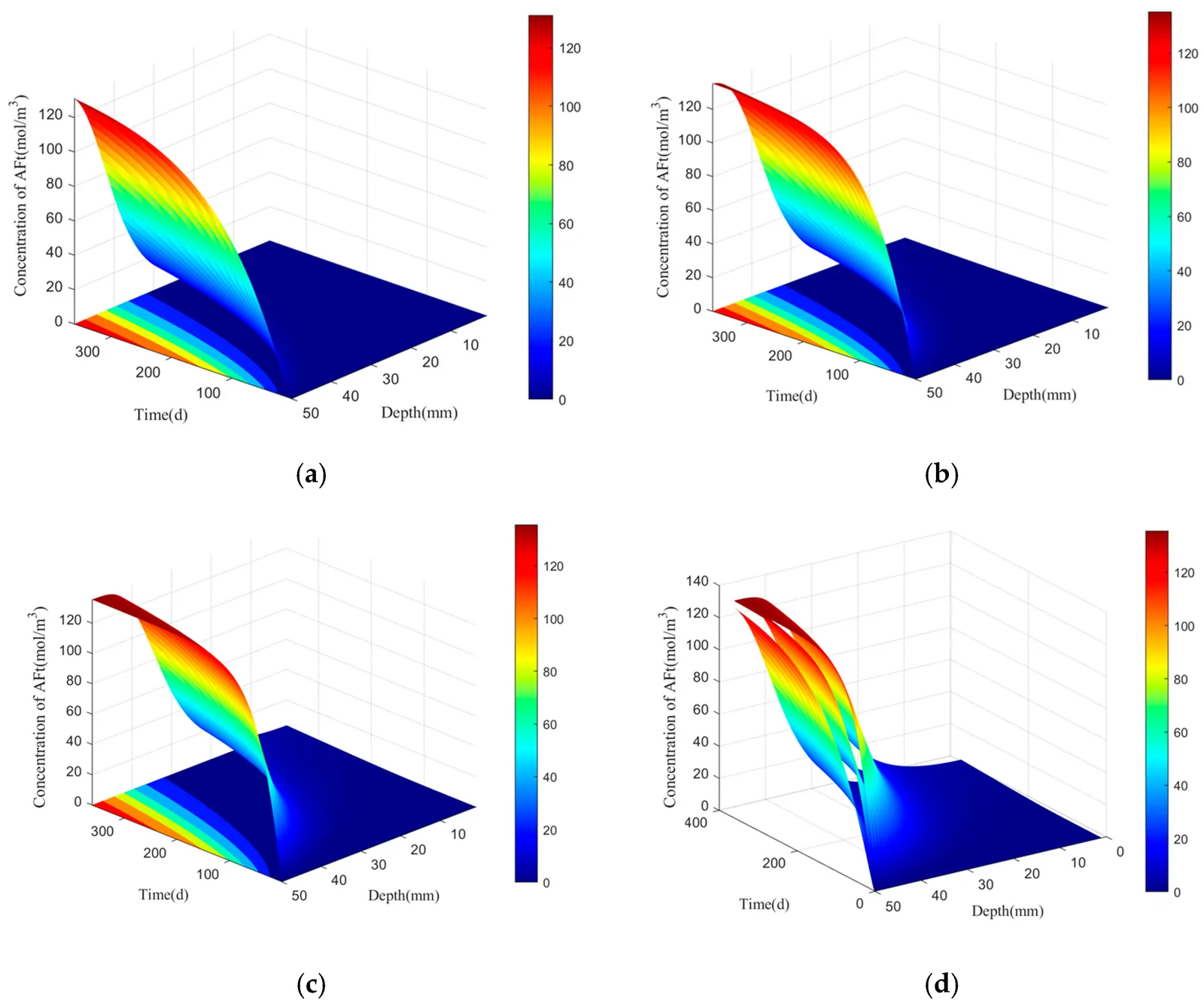
Simulated depth–time–concentration distribution of AFt at different temperatures: (**a**) 288 K; (**b**) 298 K; (**c**) 308 K; (**d**) AFt at different temperatures.

However, it should be noted that the influence of temperature on sulfate ion transport is not solely promotive. On the one hand, an increase in the diffusion coefficient facilitates the continuous penetration of sulfate ions into the concrete interior. On the other hand, the enhanced reaction kinetics accelerate the reaction between sulfate ions and calcium aluminate hydration products, promoting the formation of AFt and leading to substantial consumption of sulfate ions in the surface and near-surface regions, as shown in Figure 9 and Figure 10.

**Figure 11:**
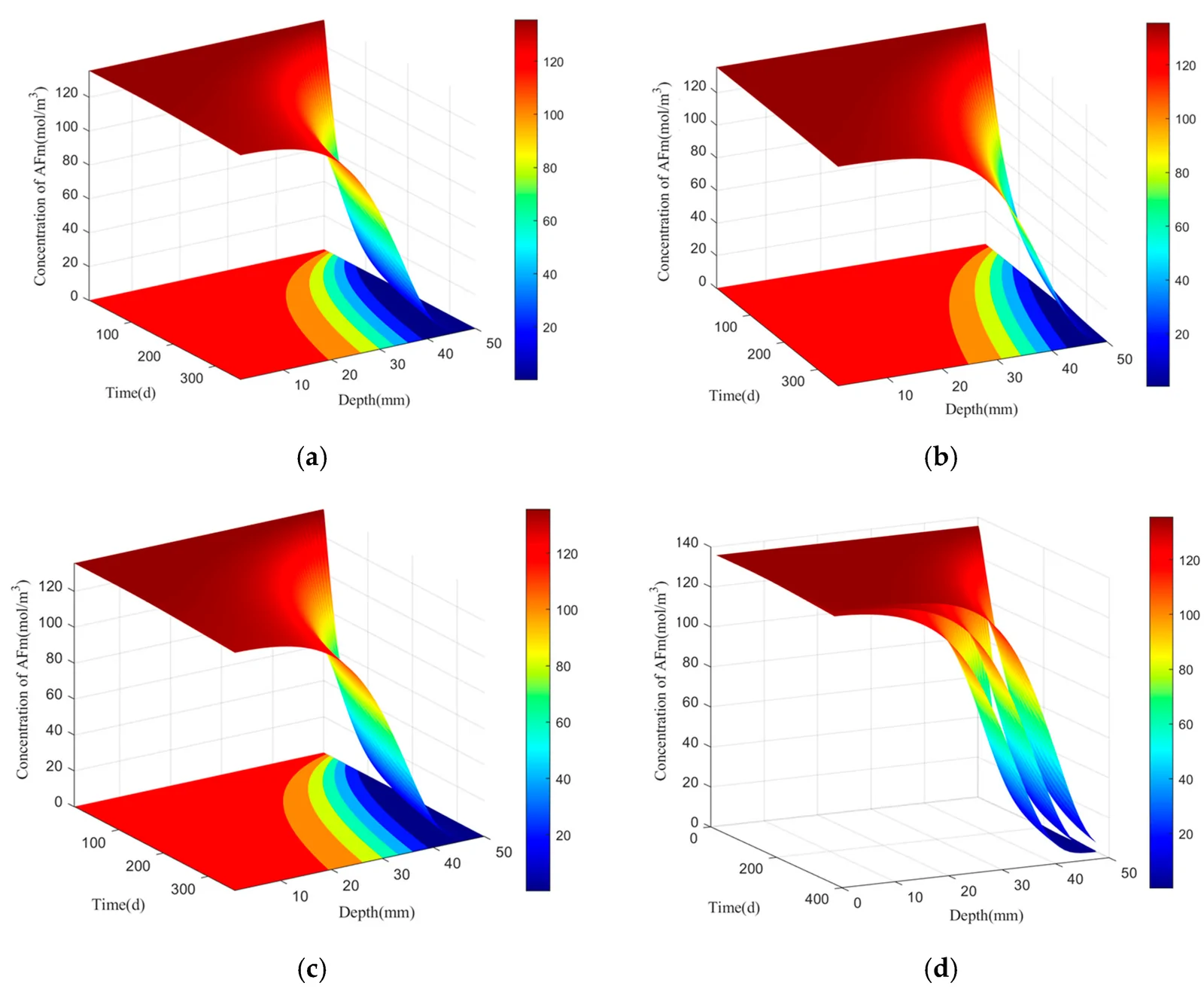
Simulated depth–time–concentration distribution of AFm at different temperatures: (**a**) 288 K; (**b**) 298 K; (**c**) 308 K; (**d**) AFm at different temperatures.

Based on Section 2.1, the initial concentration of AFm (C_AFm0_) for the *w*/*c* of 0.5 is calculated to be 135.61 mol·m^−3^. As shown in Figure 11, the AFm concentration decreases progressively within the sulfate-affected region, and the AFm depletion front moves further into the material with increasing temperature. This further confirms that elevated temperature accelerates the reaction between sulfate ions and AFm, thereby promoting AFt formation and increasing sulfate consumption. These results indicate that both chemical reactions and ionic diffusion are accelerated with increasing temperature, owing to the temperature dependence of the reaction rate constants and diffusion coefficients [53]. As the attack progresses, however, the effective regions of sulfate ion concentration under different temperature conditions gradually converge. This suggests that, over longer exposure periods, the inhibiting effect of chemical consumption on sulfate ion transport partially offsets the diffusion enhancement induced by elevated temperature, driving the system toward a dynamic balance governed by the competition between reaction consumption and diffusive supply.

### 4.3. Water-to-Cement Ratios

**Figure 12:**
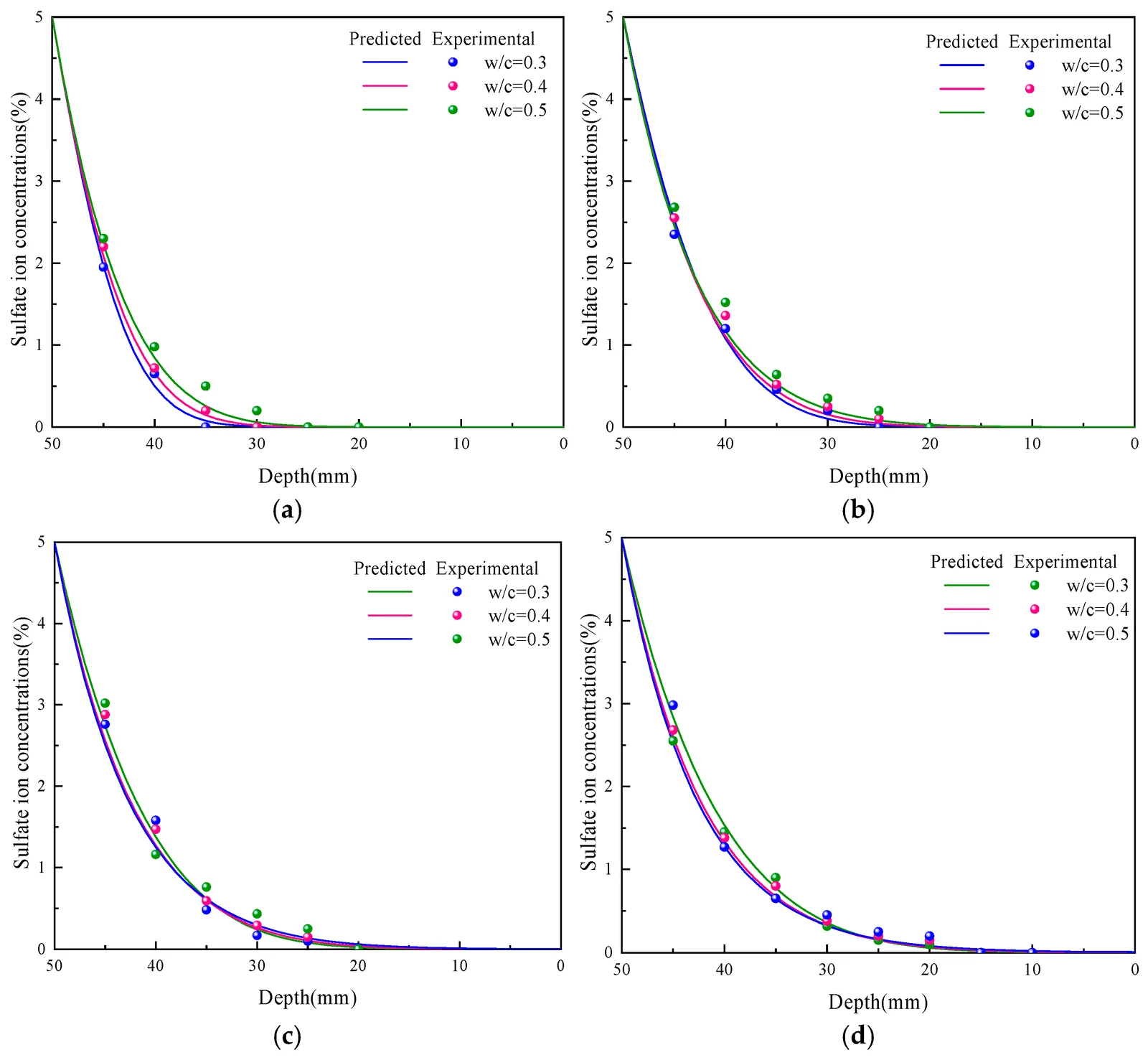
The concentration of sulfate ions at different water-to-cement ratios: (**a**) 60 days; (**b**) 120 days; (**c**) 180 days; (**d**) 240 days.

Figure 12 illustrates the sulfate ion concentration profiles in cement paste with different water-to-cement ratios (0.3, 0.4, and 0.5). The experimental values represent the mean of three replicate measurements for each water-to-cement ratio. Comparing the specimens reveals that, as the water-to-cement ratio increases, the overall sulfate ion concentration within the paste rises significantly, and the effective penetration depth becomes markedly greater. At lower water-to-cement ratios, sulfate ions are primarily concentrated near the surface, with the internal concentration decreasing sharply, indicating strong transport resistance. In contrast, at higher water-to-cement ratios, sulfate ions can penetrate deeper into the material within the same exposure period, and relatively high concentrations are maintained even at larger depths.

**Figure 13:**
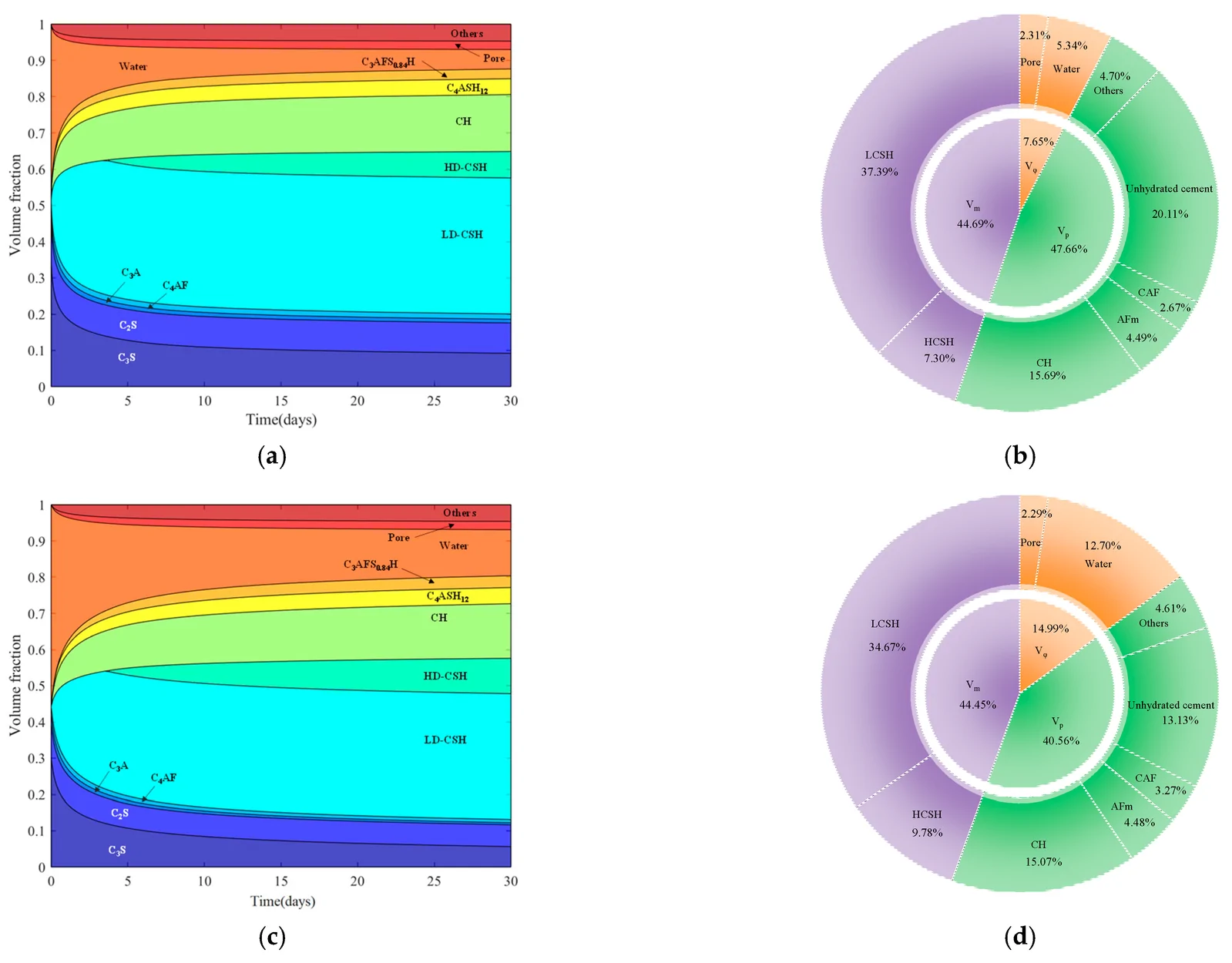
Hydration product volume fractions by the established model at different water-to-cement ratios: (**a**) Evolution of phase volume fractions in cement paste over exposure time at *w*/*c* = 0.3; (**b**) Phase volume fractions in cement paste at 28 days at *w*/*c* = 0.3; (**c**) Evolution of phase volume fractions in cement paste over exposure time at *w*/*c* = 0.4; (**d**) Phase volume fractions in cement paste at 28 days at *w*/*c* = 0.4.

This behavior is primarily attributed to the significant influence of the water-to-cement ratio on the pore structure of cement paste. As the water-to-cement ratio increases, both the volume fraction and connectivity of capillary pores are substantially enhanced, providing more favorable pathways for sulfate ion diffusion. Figure 5 and Figure 13 illustrate the hydration products of cement paste at different water-to-cement ratios, showing that the porosity increases from 7.74% to 15.0% and 23.14% for water-to-cement ratios of 0.3, 0.4, and 0.5, respectively, indicating a notably more open pore structure under high water-to-cement ratios conditions. Based on the established multiscale diffusion coefficient model, the effective diffusion coefficients of sulfate ions under different water-to-cement ratios were further calculated. The results show a clear increasing trend: as the water-to-cement ratio rises from 0.3 to 0.5, the effective diffusion coefficients are 3.73 × 10^−12^ m^2^/s, 4.94 × 10^−12^ m^2^/s and 6.83 × 10^−12^ m^2^/s respectively. These findings indicate that the microstructural changes induced by higher water-to-cement ratios directly enhance the macroscopic transport capacity of sulfate ions, allowing them to diffuse more rapidly and penetrate deeper into the cement matrix under identical exposure conditions.

**Figure 14:**
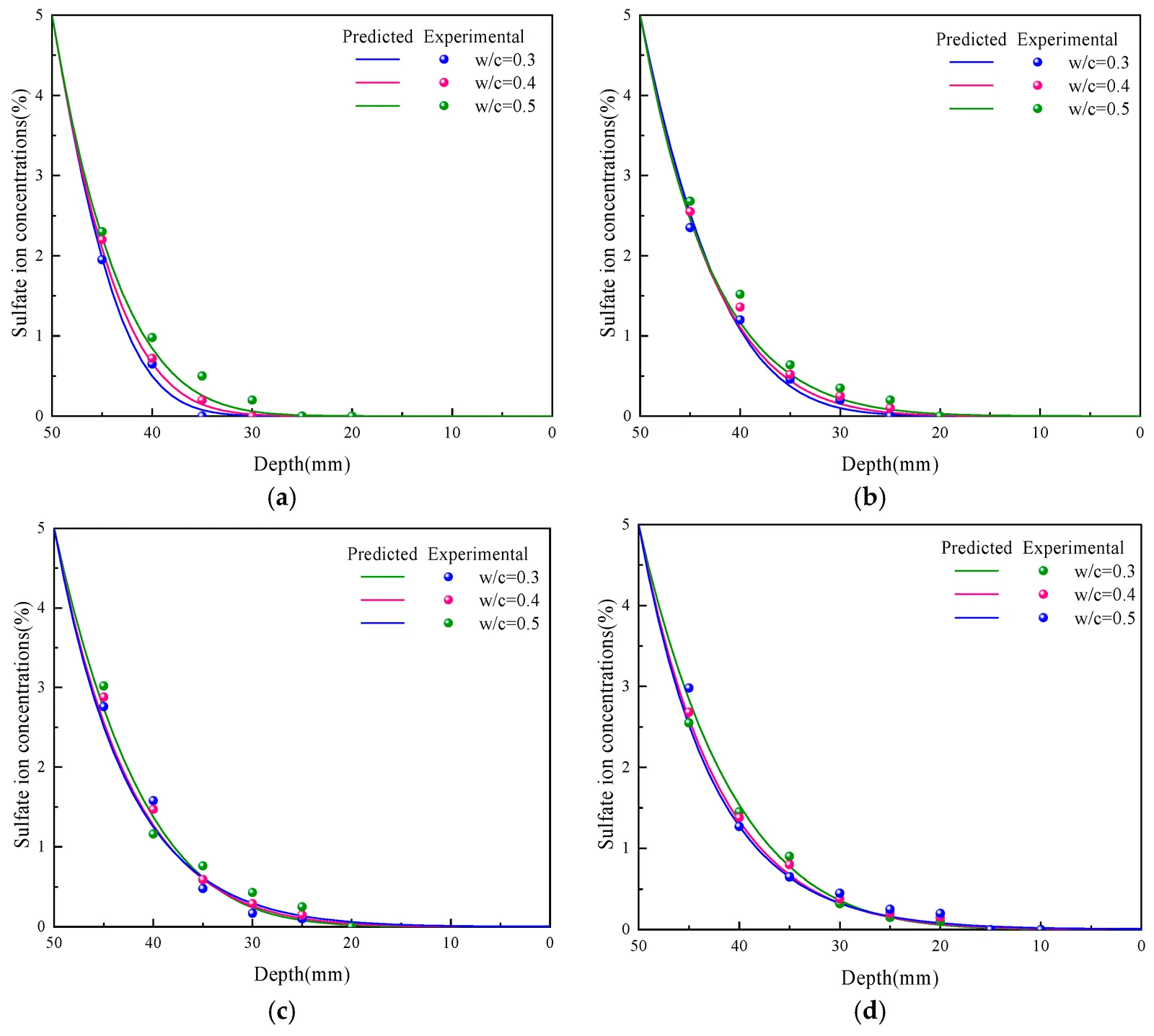
The concentration of AFt at different water-to-cement ratios: (**a**) 60 days; (**b**) 120 days; (**c**) 180 days; (**d**) 240 days.

**Figure 15:**
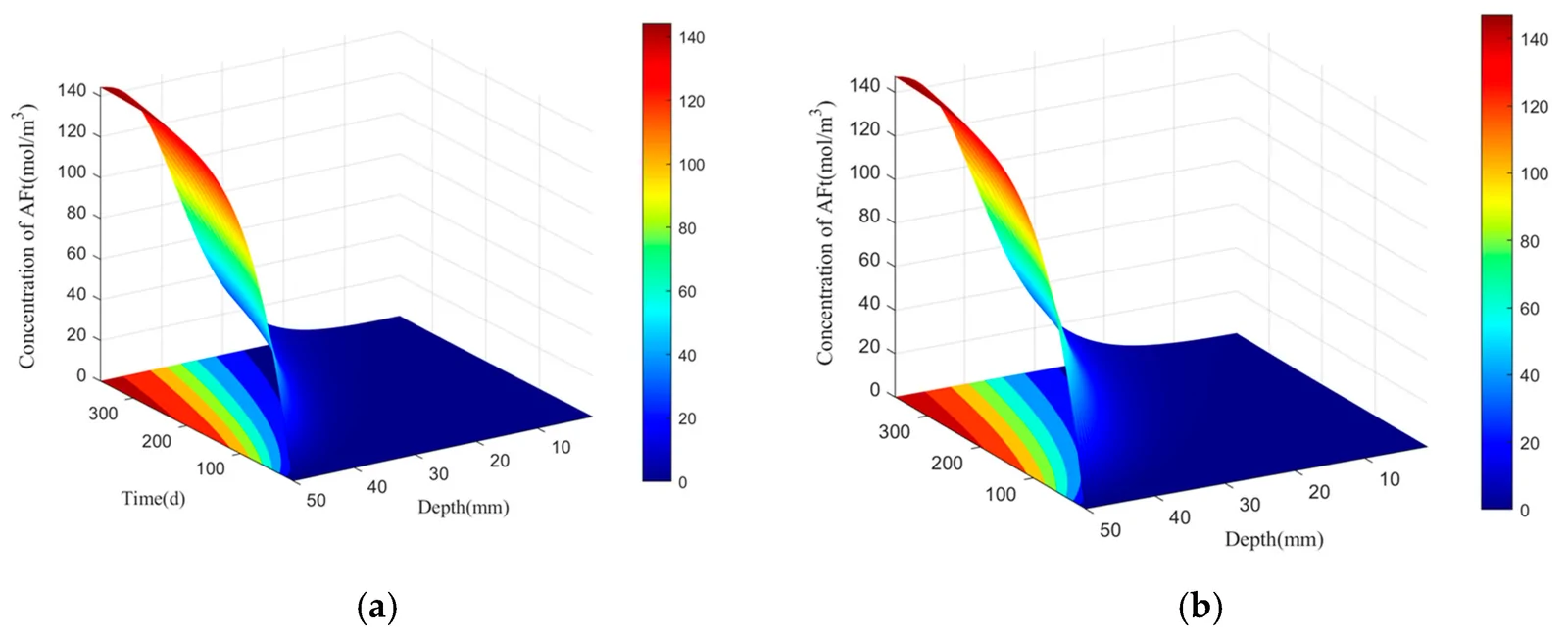

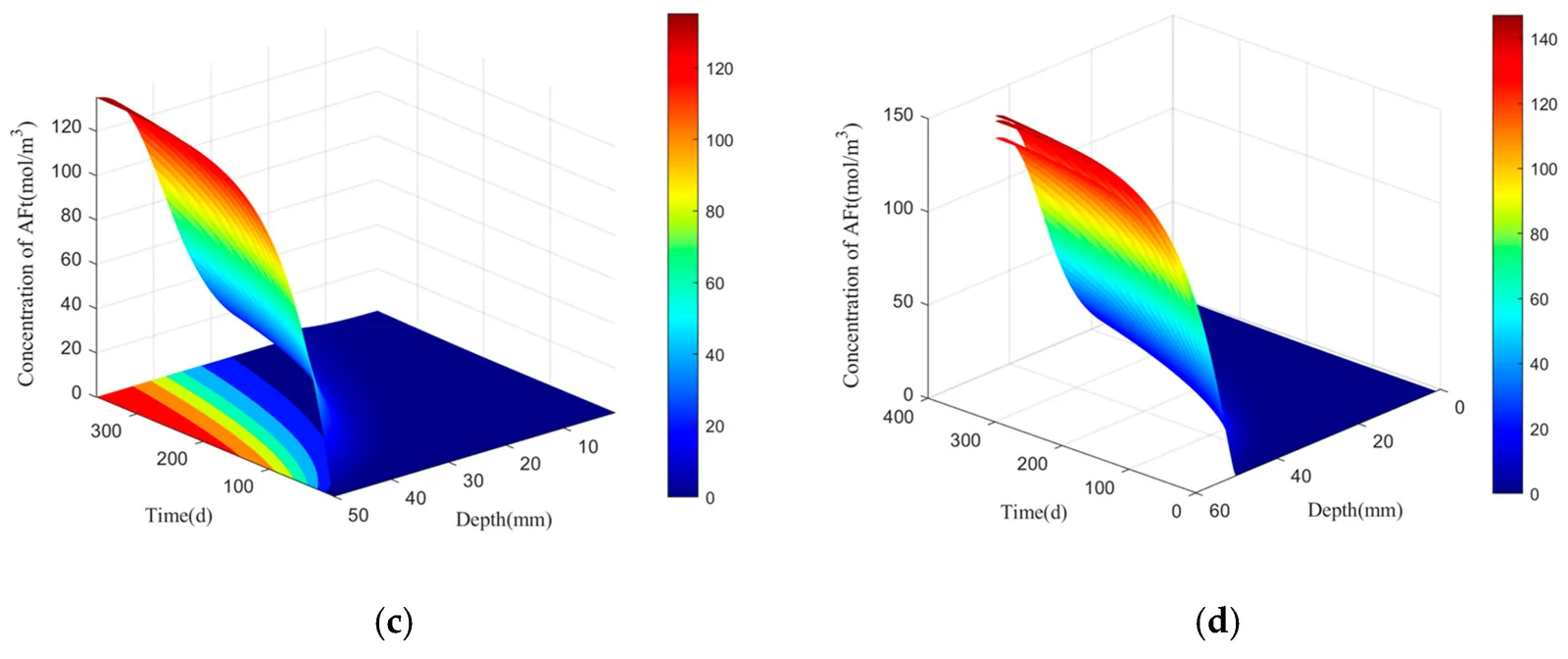
Simulated depth–time–concentration distribution of AFt at different *w*/*c*: (**a**) w/c = 0.3; (**b**) w/c = 0.4; (**c**) w/c = 0.5; (**d**) AFt at different water-to-cement ratios.

**Figure 16:**
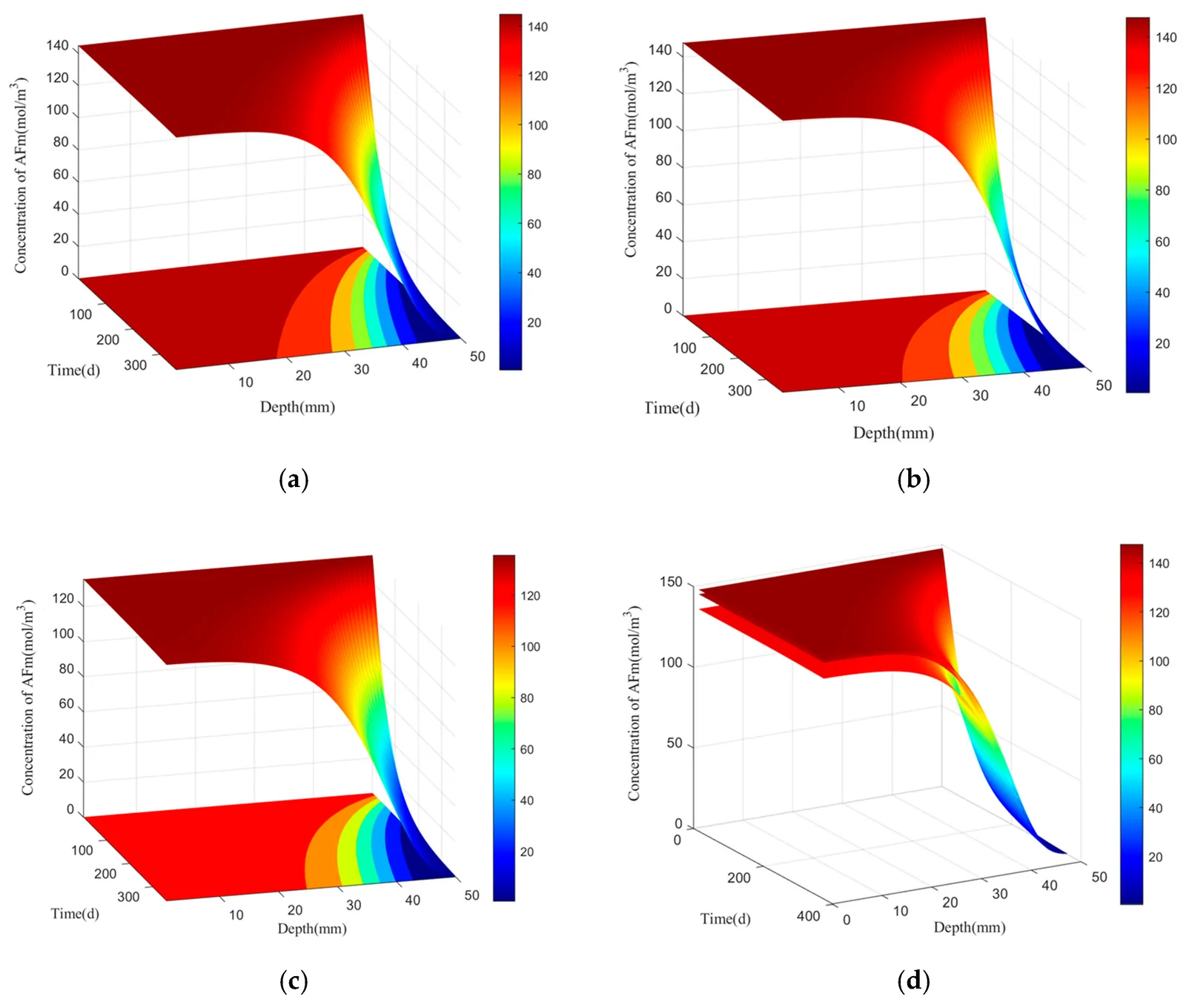
Simulated depth–time–concentration distribution of AFm at different water-to-cement ratios: (**a**) w/c = 0.3; (**b**) w/c = 0.4; (**c**) w/c = 0.5; (**d**) AFm at different water-to-cement ratios.

Figure 14 compares the simulated and experimental results of AFt formation under different water-to-cement ratios. The results show that the overall trends are in good agreement. Similar to the sulfate ion concentration profiles, both the consumption of AFm and the formation of AFt increase continuously with exposure time; however, the growth rate gradually slows during the mid-to-late stages, indicating a deceleration in the advancement of the corrosion front, as shown in Figure 15 and Figure 16. Based on Section 2.1, C_AFm0_ are calculated to be 147.64 mol·m^−3^ and 144.81 mol·m^−3^ for *w*/*c* of 0.3 and 0.4, respectively. As the water-to-cement ratio decreases, AFm consumption becomes more pronounced and AFt formation is significantly enhanced, indicating a more intense sulfate attack under lower water-to-cement ratios conditions, primarily due to the higher initial AFm content [54]. Conversely, higher water-to-cement ratios increase the effective diffusion coefficient, promoting faster migration of sulfate ions and accelerating the chemical reaction. Overall, the diffusion process is more sensitive to variations in water-to-cement ratio than the chemical reaction process.

## 5. Conclusions

This study addresses the external sulfate attack of cement-based materials by developing a coupled transport–reaction model that accounts for both ion transport and chemical reaction processes, systematically analyzing the mechanisms by which the water-to-cement ratio and temperature influence sulfate-induced degradation. The model was validated through controlled external sulfate exposure experiments, and the main findings are as follows:

(1) The model predictions of sulfate ion concentration distributions and their temporal evolution show good agreement with experimental observations. The model can accurately capture sulfate ion transport and accumulation within cement-based materials at different exposure times and effectively reflect the influence of environmental factors, such as water-to-cement ratio and temperature, on ion transport rates and cumulative effects.
(2) The water-to-cement ratio exerts a decisive influence on sulfate ion transport and the resulting degradation. As the water-to-cement ratio increases, both the capillary porosity and pore connectivity of the cementitious matrix increase significantly, leading to a higher effective diffusion coefficient and accelerating sulfate penetration. Moreover, the more porous microstructure provides favorable conditions for chemical reactions between sulfate ions and cement hydration products, enhancing the reaction extent and exacerbating microstructural deterioration. It should be noted that a lower water-to-cement ratio may result in a higher local AFt concentration near the exposed surface, whereas a higher water-to-cement ratio mainly promotes deeper sulfate penetration and thus increases the overall depth of deterioration.
(3) Temperature exhibits a pronounced coupled amplification effect on external sulfate attack. Elevated temperatures enhance sulfate diffusion not only by reducing pore solution viscosity and increasing ion mobility but also by accelerating dissolution–precipitation reactions and the kinetics of relevant mineral phase transformations, thereby increasing the reaction rate. Under higher temperature conditions, sulfate accumulation within the material is accelerated, and the reaction front advances more rapidly into the interior, resulting in greater penetration depth and more severe degradation.

In summary, the water-to-cement ratio and temperature each influenced sulfate transport and ettringite formation within the investigated conditions. These factors should therefore be represented explicitly in numerical models of external sulfate attack. After reconstructing the free sulfate mass fraction from the measured total sulfate and AFt-bound sulfate fractions, the proposed transport and reaction model reproduced the experimental concentration profiles at different immersion durations. The model provides a quantitative framework for interpreting sulfate ingress and reaction-phase evolution in cement paste within the tested parameter range. Further validation under independent exposure conditions and against expansion, cracking, and mechanical degradation data is required before the model can be applied to durability design or service-life prediction.

## Figures and Tables

**Table 1:** Constants for calculating the hydration degrees of various clinker minerals.

	C_3_S	C_2_S	C_3_A	C_4_AF
K_1_	1.5	0.5	1	0.37
N_1_	0.7	1	0.85	0.7
K_2_	0.05	0.006	0.04	0.015
K_3_	1.1	0.2	1	0.4
N_3_	3.3	5	3.2	3.7
H	1.8	1.35	1.6	1.45
E	41,570	20,785	54,040	34,087

**Table 2:** Parameters related to the hydration model.

	C_3_S	C_2_S	C_3_A	C_4_AF	*w*	*c*	C-S-H	CH
*Mx*	228.32	172.24	270.20	430.12	18	-	227.2	74
$\theta_{CSH}^{X}$	1.0	1.0	-	-	-	-	-	-
$\theta_{CH}^{X}$	1.3	0.3	-	-	-	-	-	-
$\theta_{w}^{X}$	5.3	4.3	10.0	10.75	-	-	-	-
$\theta_{V}^{X}$	−0.073	−0.077	−0.077	−0.077	-	-	-	-

**Table 3:** Chemical compositions of cement clinker.

Composition	SiO_2_	Al_2_O_3_	Fe_2_O_3_	CaO	MgO	SO_3_	K_2_O	LOI
wt.%	22.19	5.78	2.25	59.69	3.63	2.46	0.45	2.77

**Table 4:** The experiments scheme.

Group	Temperature (K)	*w*/*c*	Times (Days)
1	298	0.5	60, 120, 180, 240, 300, 360
2	288, 298, 308	0.5	60, 120, 180, 240
3	298	0.3, 0.4, 0.5	60, 120, 180, 240

## Data Availability

The data is available on request to the corresponding author.
